# Silencing arachidonate 15-lipoxygenase alleviates hypoxia-induced cognitive impairment through the mediation of phospholipase A2 group IVC–LPC18:0 to suppress ferroptosis

**DOI:** 10.1186/s43556-026-00546-w

**Published:** 2026-08-26

**Authors:** Zhen Li, Jie Gao, Jun Fu, Xi Zhang, Yiyang Zhou, Chen Li, Jutaek Nam, Hongchang Gao

**Affiliations:** 1 School of Pharmaceutical Sciences, Oujiang Laboratory (Zhejiang Lab for Regenerative Medicine, Vision and Brain Health), Institute of Metabonomics & Medical NMR, Wenzhou Medical University, Wenzhou, 325035 China; 2 College of Pharmacy, Chonnam National University, Gwangju, 61186 Republic of Korea; 3 Research and Experiment Center, Innovation Academy of Testing Technology, Wenzhou Medical University, Wenzhou, 325035 China

**Keywords:** Arachidonate 15-lipoxygenase, Phospholipase A2 group IVC, Lysophosphatidylcholine 18:0, Cognitive impairment

## Abstract

Hypoxic injury is a major contributor to cognitive impairment (CI), but the lipid metabolic mechanisms linking hypoxia (HYP) to hippocampal ferroptosis remain understood. This study investigated whether the arachidonate 15-lipoxygenase-cytosolic phospholipase A2 group IVC-lysophosphatidylcholine 18:0 (ALOX15-PLA2G4C-LPC18:0) axis contributes to HYP-induced hippocampal ferroptosis and CI. Morris water maze testing, Nissl staining, and NeuN immunofluorescence showed that HYP impaired spatial learning and memory and induced hippocampal neuronal loss in mice. Semi-targeted LC–MS/MS lipidomics revealed marked phospholipid/lysophospholipid (PL/LPL) remodeling, characterized by increased phosphatidylcholine and phosphatidylethanolamine species and decreased lysophosphatidylcholine species, particularly LPC18:0. Ferroptosis-related biochemical assays and transmission electron microscopy further demonstrated glutathione (GSH) depletion, malondialdehyde (MDA) accumulation, glutathione peroxidase 4 (GPX4) downregulation, and mitochondrial abnormalities. GEO dataset analysis, qRT-PCR, and Western blotting identified ALOX15 as a HYP-responsive ferroptosis-associated regulator. Hippocampal AAV-mediated Alox15 knockdown improved cognitive performance, preserved hippocampal neurons, suppressed lipid peroxidation, and partially restored PL/LPL homeostasis. In HYP-exposed SH-SY5Y cells, genetic and pharmacological experiments further supported the contribution of ALOX15 to ferroptosis-related injury. RNA sequencing, promoter-reporter assays, and gain- and loss-of-function experiments suggested that ALOX15 suppressed PLA2G4C expression partly through MAFK-dependent transcriptional modulation. PLA2G4C restoration increased LPC18:0 production, whereas LPC18:0 supplementation attenuated ferroptosis-related injury and improved hippocampal pathology and cognitive performance in HYP-exposed mice. Collectively, these findings support the ALOX15-PLA2G4C-LPC18:0 axis as a potential lipid-remodeling mechanism underlying HYP-induced hippocampal ferroptosis and highlight ALOX15 inhibition and LPC18:0 restoration as potential strategies for mitigating HYP-induced CI.

**Supplementary Information:**

The online version contains supplementary material available at 10.1186/s43556-026-00546-w.

## Introduction

The mammalian brain represents only about 2% of total body weight but consumes nearly 20% of the body’s oxygen supply, making it highly vulnerable to oxygen insufficiency [1, 2]. Hypoxia (HYP) occurs in high-altitude environments, extreme climates, and specific occupational settings, such as cavern emergency rescue, law enforcement, and military deployment, and can negatively affect human health and cognitive function [3]. In addition, clinical conditions, including infarction, anemia, carbon monoxide poisoning, and lung disease, are often accompanied by HYP and may cause varying degrees of cognitive impairment [4–8]. Notably, nearly half of postnatal infants exposed to intrauterine HYP show long-term sequelae associated with abnormal cognitive development [9].

HYP is also implicated in Alzheimer’s disease (AD), Parkinson’s disease (PD), and other age-related neurodegenerative disorders, prompting extensive investigation into the mechanisms of HYP-induced brain dysfunction [2, 10]. Early studies using Caenorhabditis elegans showed that hypoxia disturbed neuronal wiring through a hif1a-dependent pathway involving the Eph receptor VAB-1 [11]. These early findings established that HYP can directly perturb neuronal structure and connectivity, prompting further investigation into cellular and molecular mechanisms of HYP-induced neural injury. In particular, emerging evidence indicates that HYP can trigger iron-dependent ferroptotic cascades in hippocampal neurons, linking oxygen deprivation to lipid peroxidation and neuronal loss. Over the years, researchers have investigated HYP-induced brain dysfunction through approaches focusing on glial cell defense, oxygen imaging techniques, and molecular mechanisms, yet effective and curative treatment strategies have remained elusive [12, 13]. A deeper understanding of HYP pathogenesis is needed to develop effective preventive and therapeutic strategies for HYP-associated neurological disorders.

HYP-mediated cellular injury is mechanistically linked to iron dyshomeostasis and ferroptosis; this has been validated in adipose tissue [14] and neonatal murine hippocampus [15]. These findings highlight the need to define the molecular mechanisms of HYP-triggered ferroptotic cascades. Ferroptosis is an iron-dependent form of regulated cell death driven by the peroxidation of polyunsaturated fatty acid that generates lipid hydroperoxides through peroxyl radical intermediates [16, 17]. Central to this process is arachidonate 15-lipoxygenase (ALOX15), an enzyme that catalyzes the generation of phospholipids (PLs) peroxidation products essential for ferroptosis. ALOX15 has multiple roles not only in cellular differentiation but also pathogenesis of various diseases [18], and thus has been extensively investigated in therapeutic contexts for PD, glioblastoma [19], and colorectal carcinogenesis [20], suggesting its potential therapeutic relevance.

Several key questions therefore remain unresolved. First, although HYP is associated with ferroptosis-related injury, whether ALOX15 functions as a driver of ferroptotic damage in hippocampal neurons under HYP is still unknown. Second, previous ALOX15 studies have mainly focused on tumors, inflammatory disorders, or classical neurodegenerative diseases, whereas its role in HYP-induced neural injury and learning/memory deficits has not been sufficiently defined. Third, the lipid metabolic events downstream of ALOX15 activation remain poorly understood, particularly whether ALOX15 regulates ferroptosis through remodeling of PL/lysophospholipid (LPL) metabolism. Finally, it remains unknown whether modulation of an ALOX15-dependent lipid metabolite can rescue hippocampal ferroptosis and improve HYP-induced cognitive impairment (CI). In this study, CI was defined as measurable deficits in learning and memory, evaluated by the Morris water maze (MWM) test together with histological assessment of hippocampal injury. Thus, this study addresses the gap linking HYP-induced ALOX15 activation, lipid remodeling, ferroptotic injury, and cognitive dysfunction.

In this study, we investigated whether ALOX15 contributes to HYP-induced hippocampal ferroptosis and CI and explored the downstream lipid metabolic mechanism. We hypothesized that HYP-induced ALOX15 activation promotes ferroptotic injury by disrupting PLA2G4C-mediated PL/LPL metabolism and lysophosphatidylcholine 18:0 (LPC18:0) homeostasis. To test this hypothesis, we combined LC–MS/MS-based lipidomics, bioinformatic screening of ferroptosis-related genes from GEO datasets, hippocampal AAV-mediated Alox15 knockdown, MWM behavioral assessment, histological evaluation of hippocampal injury, and molecular assays including Western blot, qRT-PCR, and ferroptosis-related biochemical analyses. This study aimed to clarify the role of the ALOX15–PLA2G4C–LPC18:0 axis in HYP-induced hippocampal ferroptosis and provide mechanistic insight into HYP-induced CI.

## Results

### Hypoxia induces cognitive impairment and neuronal loss in mice

**Fig. 1:**
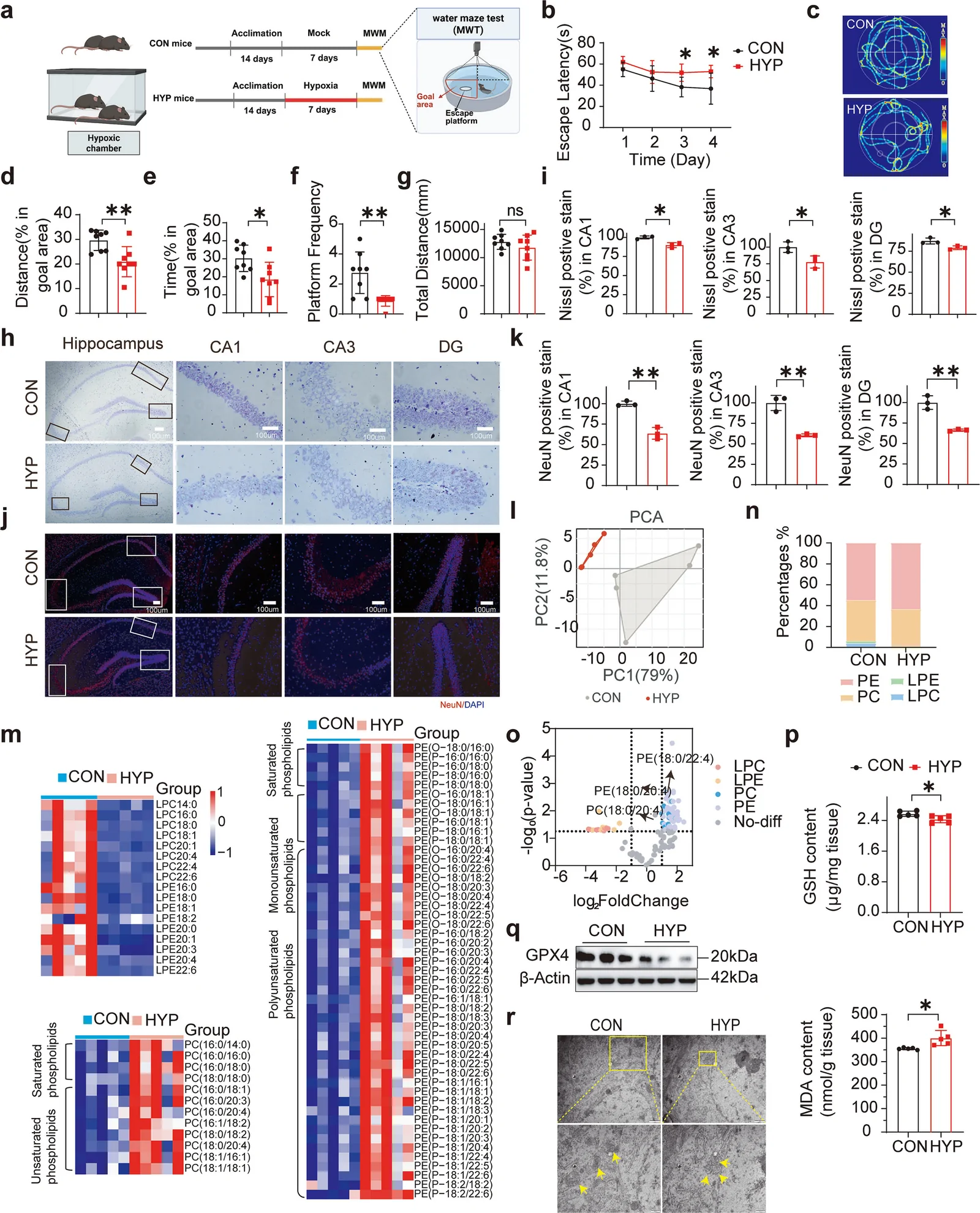
Hypoxia (HYP) induces cognitive impairment and neuronal loss in mice with phospholipids/lysophospholipids disorders and ferroptosis in the hippocampus. **a** Experimental timeline for the control and HYP exposure groups. **b** The escape latency of CON and HYP mice during the positioning navigation test (*n* = 8). **c** The trajectories, (**d**) the distance percentages, (**e**) the time in the goal area, (**f**) the number of crossings over the original platform location, and (**g**) the total distance covered by the mice in the spatial exploration swimming test (*n* = 8). **h** Representative images of Nissl body staining. Nissl-positive neuronal cell bodies are stained blue-purple. Boxes in the whole-hippocampus images indicate the regions shown at higher magnification. Scale bars, 100 μm. **i** Quantitative results of the Nissl staining in the mouse hippocampal CA1, CA3, and DG regions (*n* = 3). **j** Representative images of NeuN staining. NeuN-positive neurons are shown in red, and nucleus stained with DAPI are shown in blue. Boxes indicate the regions shown at higher magnification. Scale bars, 100 μm. **k** Quantitative results of the NeuN staining in the mouse hippocampal CA1, CA3, and DG regions (*n* = 3). **l** PCA analysis of the metabolic pattern changes in the hippocampus. **m** The heatmap showing relative levels of differential lipid metabolites in the hippocampus; red indicates higher relative levels and blue indicates lower relative levels (*n* = 5). **n** Stacked bar chart of lipid metabolite distribution (*n* = 5). **o** Volcano plot analysis of the lipid metabolites. Colored points indicate significantly altered lipid species, whereas gray points indicate non-significantly altered lipid species. **p** Hippocampal MDA and GSH levels in CON mice and mice exposed to hypoxia for 14 days (*n* = 5). **q** Representative Western blot images of GPX4 protein expression in the hippocampal tissues of mice (*n* = 3). **r** Transmission electron microscopy (TEM) images of the mice hippocampus. Scale bar, 1 µm and 500 nm. Student’s t-test was used to determine the significant differences. Significance was determined at a *p*-level of * *p* < 0.05; ** *p* < 0.01; ns = no significance

To evaluate the effect of HYP on learning and memory, mice were subjected to the MWM test after HYP exposure (Fig. 1a). During training, HYP-exposed mice showed prolonged escape latency compared with controls (Fig. 1b). In the probe trial, HYP reduced swimming path length and time spent in the target quadrant (Q-III), as well as the number of platform crossings, while total swimming distance remained comparable between groups (Fig. 1c–g). Because CI is closely associated with hippocampal neuronal damage, Nissl and immunofluorescence (IF) staining were performed. HYP markedly reduced neuronal density in the CA1, CA3, and dentate gyrus (DG) regions, accompanied by weakened Nissl staining and partial dissolution of Nissl bodies (Fig. 1h, i). Consistently, NeuN IF staining indicated hippocampal neuronal loss after HYP exposure (Fig. 1j, k). These findings suggest that HYP impairs spatial learning and memory and is accompanied by neuronal injury in key hippocampal subregions.

### Hypoxia induces phospholipid/lysophospholipid metabolic disturbance and ferroptosis-related features in the hippocampus

HYP is a well-documented disruptor of PL and LPL metabolism [21, 22]. To determine whether HYP induces hippocampal lipid metabolic remodeling and ferroptosis-related injury, we performed semi-targeted lipidomic analysis of PLs and LPLs, followed by biochemical and ultrastructural validation. Principal component analysis (PCA), an unsupervised method used to visualize global metabolic differences between groups, showed a clear separation between normoxic and HYP hippocampal lipid profiles (Fig. 1l). Heatmap analysis further revealed marked HYP-associated increases in phosphatidylcholines (PCs) and phosphatidylethanolamines (PEs), together with reductions in LPCs and lysophosphatidylethanolamines (LPEs) (Fig. 1m). Among these lipid classes, PEs represented the dominant PL subclass altered in the hippocampus after HYP exposure (Fig. 1n). Notably, PE-18:0/C20:4 and PE-18:0/C22:4, which are considered ferroptosis-associated phospholipid species, showed prominent changes under HYP (Fig. 1o).​

To further assess whether these lipid alterations were accompanied by ferroptosis-related damage, we measured glutathione (GSH), malondialdehyde (MDA), GPX4, and mitochondrial morphology. GSH is an antioxidant molecule involved in cellular redox defense, whereas MDA is a lipid peroxidation product reflecting oxidative membrane damage. HYP exposure decreased GSH levels and increased MDA levels in hippocampal tissues (Fig. 1p). In addition, GPX4, a key antioxidant enzyme that suppresses ferroptosis by reducing lipid peroxides, was downregulated after HYP exposure (Fig. 1q). Transmission electron microscopy (TEM) showed smaller and ruptured mitochondria with reduced cristae in hippocampal neurons of the HYP group (Fig. 1r). Together, these results indicate that HYP induces PL/LPL metabolic disturbance and ferroptosis-related biochemical and mitochondrial abnormalities in the hippocampus, which may contribute to hippocampal degeneration.

To assess the contribution of ferroptosis to HYP-induced CI, mice received the ferroptosis inhibitor ferrostatin-1 (Fer-1) by intraperitoneal injection every other day. Fer-1 improved MWM performance in HYP-exposed mice (Fig. S1a–f), suggesting that ferroptosis contributes to HYP-associated hippocampus injury.

### Arachidonate 15-lipoxygenase is a ferroptosis-associated candidate upregulated in the hippocampus after hypoxia

**Fig. 2:**
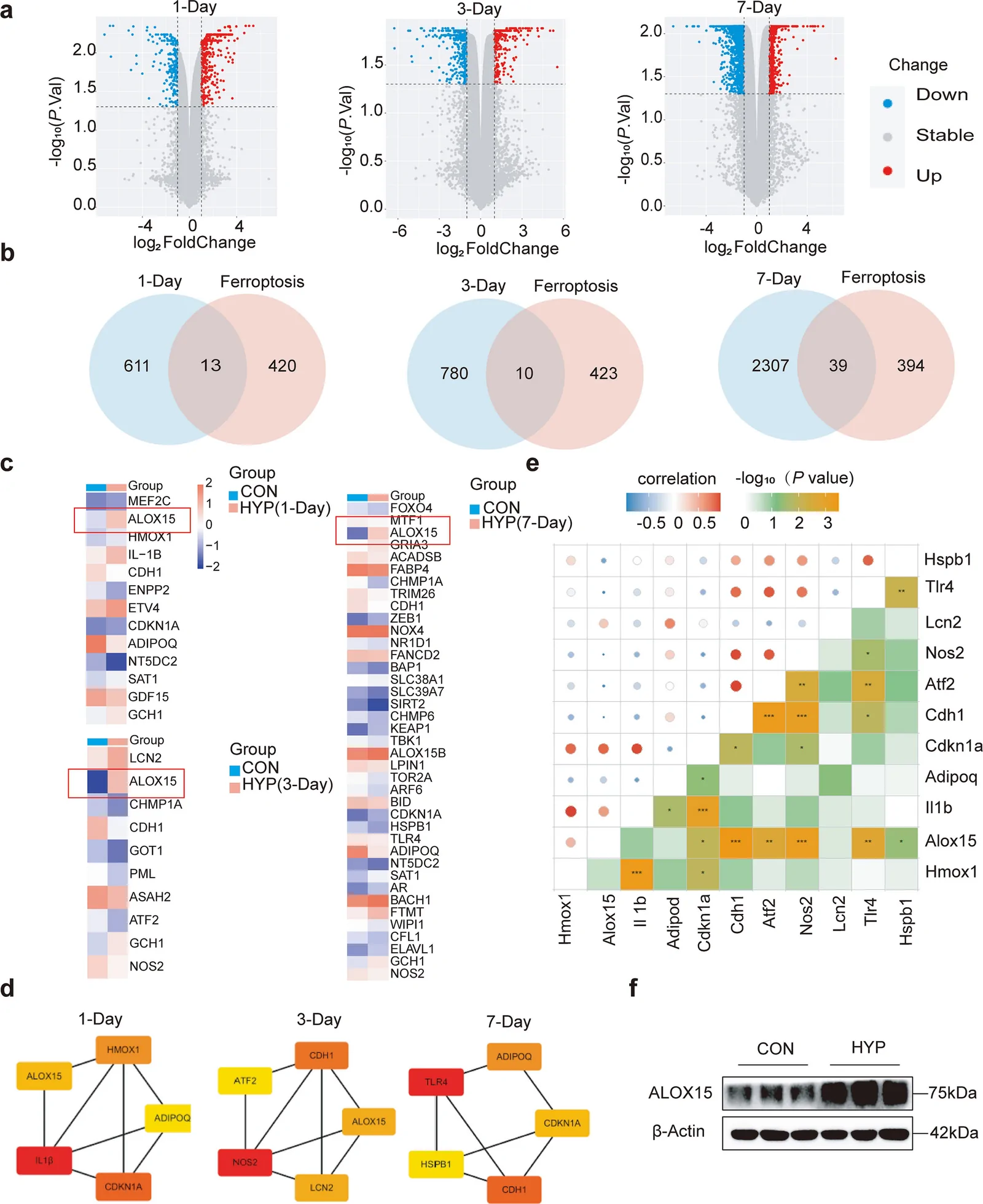
Arachidonate 15-lipoxygenase is the most significantly upregulated ferroptosis-associated factor in the hippocampus of hypoxia mice. **a** Volcano plot of differentially expressed genes (DEGs) in GSE66287. **b** Venn diagram of DEGs and ferroptosis-related genes (FRGs). **c** The dataset heatmap of critical FRGs. The expression levels of genes, ranging from high to low, are depicted by colors from red to blue, respectively. **d** PPI network diagram of central node genes based on the relationships. **e** Correlation heatmap of central node genes. Red represents a positive correlation; blue represents a negative correlation between two genes. The -log_10_(*p*-value) numbers ranging from high to low, are depicted by colors from yellow to green, respectively. **f** Representative western blot images of ALOX15 protein expression in the hippocampal tissues of mice (*n* = 3). Student’s t-test was used to determine the significant differences. Significance was determined at a *p*-level of ** *p* < 0.01

To identify molecular regulators of HYP-related ferroptosis, hippocampal transcriptomic data from HYP-exposed rats were analyzed using the GEO dataset. Using adjusted *p*-value < 0.05 and |log_2_FC|> 1 as thresholds, 624, 790, and 2346 differentially expressed genes (DEGs) were identified in the 1-day, 3-day, and 7-day HYP groups, respectively (Fig. 2a). The 7-day HYP group showed the strongest transcriptional alteration, suggesting progressive molecular remodeling under HYP. Intersecting DEGs with 433 ferroptosis-related genes (FRGs) identified 13, 10, and 39 candidate ferroptosis-related genes at 1, 3, and 7 days, respectively (Fig. 2b). Heatmap analysis showed consistent Alox15 upregulation across time points (Fig. 2c). Protein–protein interaction (PPI) analysis identified *Alox15* among the top hub genes, with strong correlations with other candidates, especially *Cdh1* and *Nos2* (Fig. 2d, e). qRT-PCR further validated selected ferroptosis-related genes in rat hippocampal tissues (Fig. S2), and Western blot indicated increased ALOX15 protein expression after HYP (Fig. 2f). These findings identify ALOX15 as a ferroptosis-associated candidate gene for subsequent functional validation in HYP-induced hippocampal injury.

### Silencing arachidonate 15-lipoxygenase protects the hippocampus against hypoxia-induced injury in mice

**Fig. 3:**
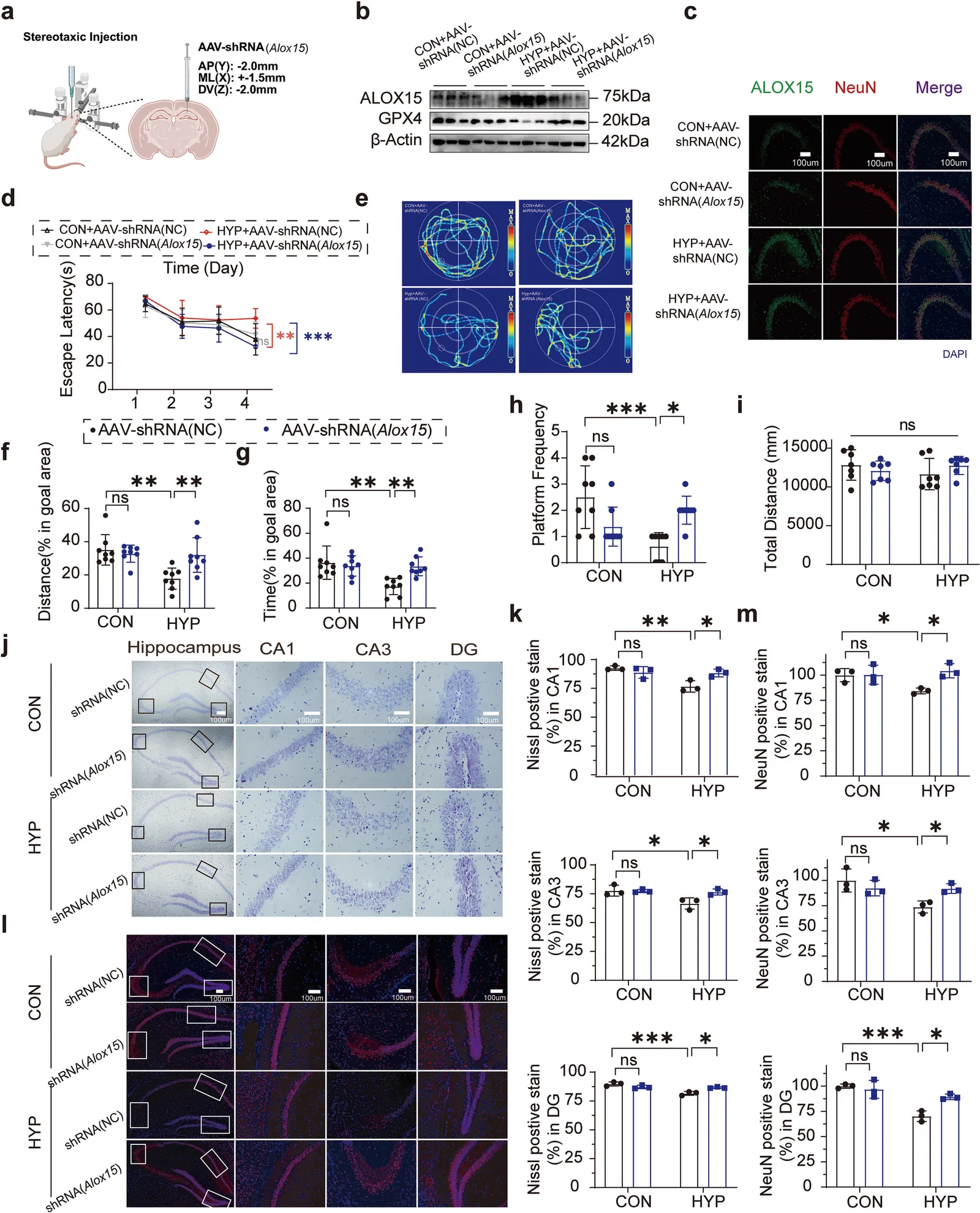
Silencing arachidonate 15-lipoxygenase protects the hippocampus against hypoxia-induced injury in mice. **a** Stereotactic modeling site in mice. **b** Representative Western blot images of ALOX15, GPX4 protein expression (*n* = 3). **c** Fluorescence staining of ALO15 and NeuN in the hippocampus of mice. Scale bars, 100 μm. **d** The escape latency of the AAV-shNC or AAV-sh*Alox15*-treated mice with or without HYP during the positioning navigation test (*n* = 8). **e** The trajectories, (**f**) the distance percentages, (**g**) the time in the goal area, (**h**) the number of crossings over the original platform location, and (**i**) the total distance of exploration by the mice in the spatial exploration swimming test (*n* = 8). **j** Representative images of Nissl body staining. Nissl-positive neuronal cell bodies are stained blue-purple. Boxes in the whole-hippocampus images indicate the regions shown at higher magnification. Scale bars, 100 μm. **k** Quantitative results of the Nissl staining in the hippocampal CA1, CA3, and DG regions of mice (*n* = 3). **l** Representative images of NeuN staining. NeuN-positive neurons are shown in red, and nucleus stained with DAPI are shown in blue. Boxes indicate the regions shown at higher magnification. Scale bars, 100 μm. **m** Quantitative results of the NeuN staining in the hippocampal CA1, CA3, and DG regions of mice. (*n* = 3). Data are presented as the mean ± SD of at least three independent experiments. The behavioral changes and quantitative results of the staining were analyzed by one-way ANOVA with Tukey’s post hoc test. Significance was determined at a *p*-level of * *p* < 0.05; ** *p* < 0.01; *** *p* < 0.001; ns = no significance

Given the potential role of ALOX15 in HYP-induced injury, AAV-shRNA targeting Alox15 was bilaterally delivered into the hippocampus to reduce local ALOX15 expression (Fig. 3a). Western blot indicated effective ALOX15 knockdown (Fig. 3b). ALOX15/NeuN co-staining further showed that HYP increased ALOX15 fluorescence in NeuN-positive hippocampal regions, whereas AAV-sh*Alox15* reduced ALOX15 expression in these neuronal layers under HYP conditions (Fig. 3c). These data support local ALOX15 reduction in hippocampal neuronal regions, although non-neuronal contributions cannot be excluded because the AAV vector was not cell-type specific. Under normoxic conditions, *Alox15* knockdown did not affect MWM performance. However, under HYP, hippocampal *Alox15* knockdown improved spatial memory, as shown by increased target quadrant time and distance and more platform crossings, without changing total swimming distance (Fig. 3d–i). Consistently, *Alox15* knockdown increased Nissl body density and NeuN immunostaining in the hippocampus (Fig. 3j–m), suggesting reduced HYP-induced neuronal injury. These findings suggest that ALOX15 may contribute to HYP-induced CI and that ALOX15 inhibition may have potential therapeutic relevance.

**Fig. 4:**
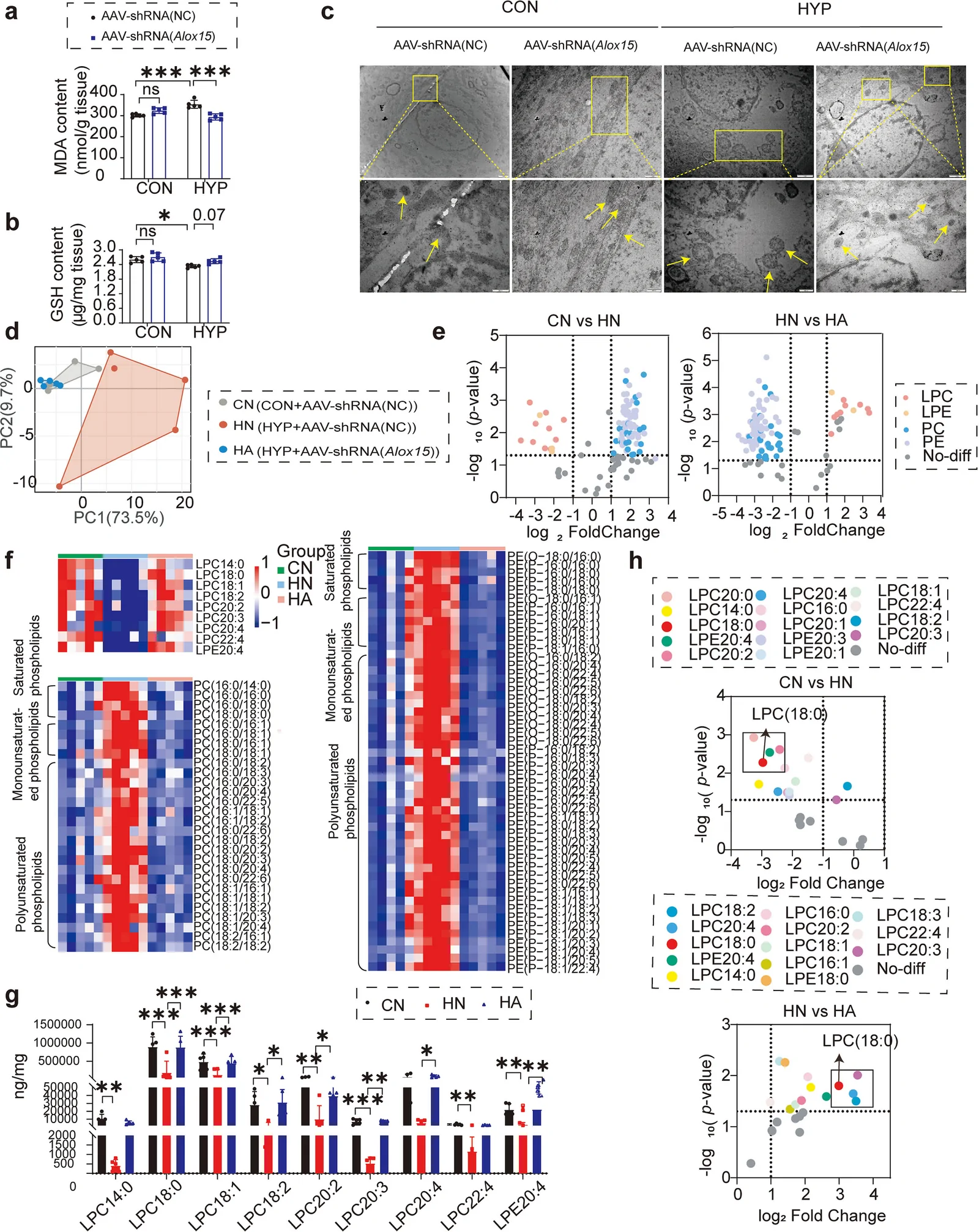
Silencing arachidonate 15-lipoxygenase protects the hippocampus against hypoxia-mediated phospholipids/lysophospholipids disorders and ferroptosis. **a**, **b** Changes in MDA and GSH levels in hippocampal tissues from CON and HYP mice injected with either control AAV-shRNA [AAV-shRNA(NC)] or AAV-shRNA targeting *Alox15* [AAV-shRNA(*Alox15*)]. (*n* = 5). **c** Representative images of mitochondrial morphological changes in the hippocampus of the AAV-shNC or AAV-sh*Alox15*-treated mice with or without HYP. **d** PCA analysis of metabolic pattern changes. Each symbol represents one biological sample, and the shaded regions indicate the distribution of samples within each group (*n* = 5). **e** Volcano plot of the lipid metabolites from the CN, HN and HA mice. Red points indicate significantly increased metabolites, blue points indicate significantly decreased metabolites, and gray points indicate metabolites without significant changes. **f** A heatmap of the relative levels of lipid metabolites in the hippocampus. Red indicates a higher relative abundance, whereas blue indicates a lower relative abundance (*n* = 5). **g** A histogram of the relative levels of LPLs in the hippocampus of mice (*n* = 5). **h** Volcano plot of the different LPL levels. Data are presented as the mean ± SD of at least three independent experiments. The differences among groups were analyzed using one-way ANOVA with Tukey’s post hoc test. Significance was determined at a *p*-level of * *p* < 0.05; ** *p* < 0.01; *** *p* < 0.001; ns = no significance

Given the observed association between ferroptosis-related injury and ALOX15 upregulation in HYP-induced CI, we investigated whether ALOX15 modulates PL/LPL metabolism and ferroptosis. The results showed that hippocampal *Alox15* knockdown attenuated HYP-induced ferroptosis-related changes, as indicated by reduced MDA and restored GSH and GPX4 levels (Fig. 4a, b). TEM analysis also showed alleviation of HYP-induced mitochondrial damage, including mitochondrial rupture and cristae loss (Fig. 4c). Lipidomic analysis revealed clear separation between control and HYP groups, indicating marked PL/LPL remodeling under HYP (Fig. 4d). *Alox15* silencing shifted the HYP lipid profile toward that of controls (Fig. 4d, e), reversed HYP-induced increases in PC and PE, and restored the reduction of LPC and LPE species (Fig. 4f, g). Targeted analysis showed that LPC18:0, LPC20:0, and LPC20:4 were markedly depleted under HYP, whereas *Alox15* knockdown preferentially restored LPC18:0, LPC20:3, and LPC20:4, with LPC18:0 emerging as a key lipid species associated with both HYP injury and Alox15 knockdown response (Fig. 4h). These findings suggest that ALOX15 contributes to HYP-induced CI by regulating ferroptosis-related injury and PL/LPL metabolic remodeling.

### Arachidonate 15-lipoxygenase knockdown protects SH-SY5Y cells from hypoxia-mediated phospholipid/lysophospholipid disturbance and ferroptosis-related injury

**Fig. 5:**
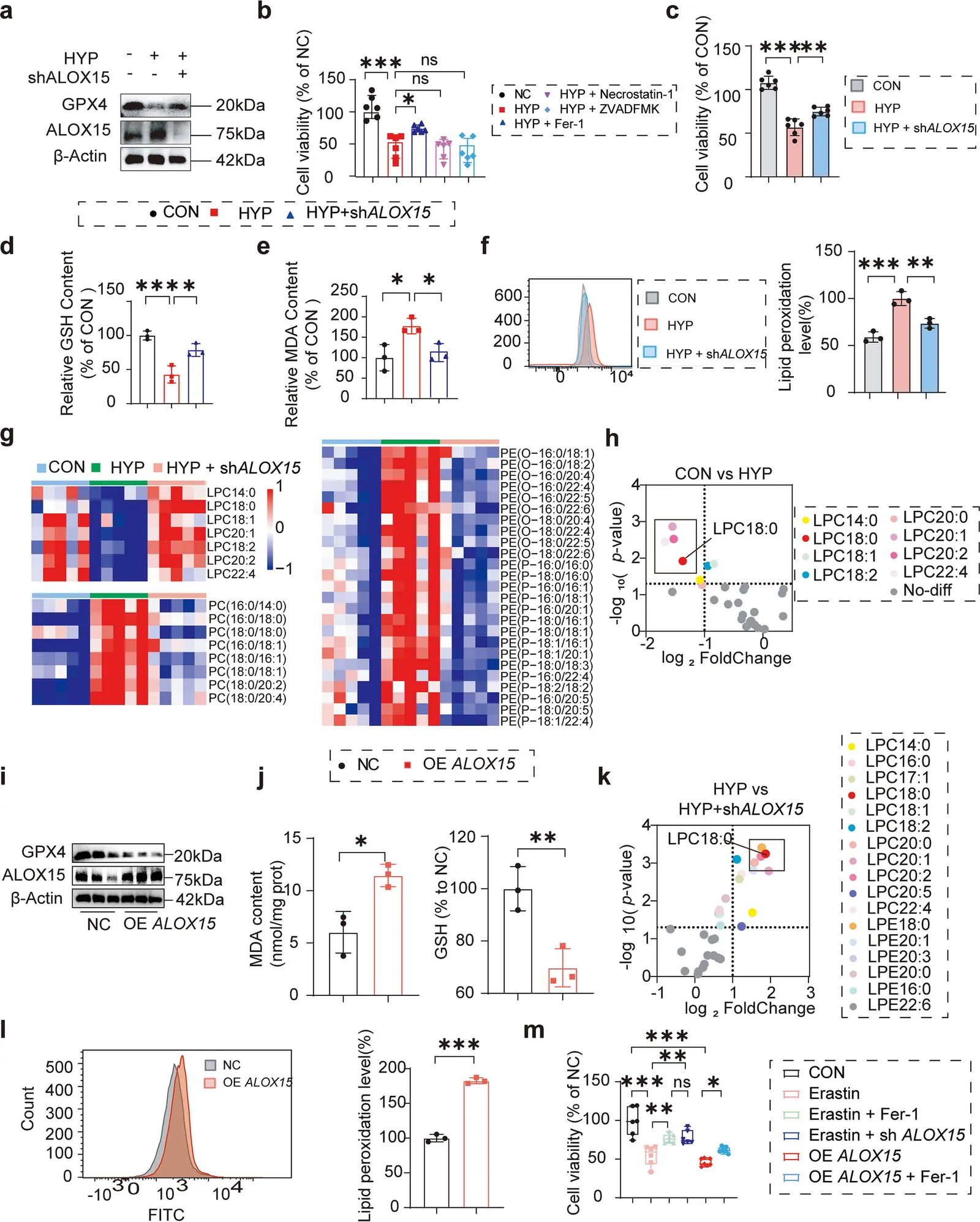
Knockdown of arachidonate 15-lipoxygenase confers the protection from hypoxia-mediated phospholipid/lysophospholipid disorders and ferroptosis. **a** Representative Western blot images of ALOX15, GPX4 protein expression (*n* = 3). **b** SH-SY5Y cells was pre-incubated with ferrostatin-1 (10 μM), necrostatin-1 (10 μM), or Z-VAD-FMK (10 μM) for 12 h and then exposed to HYP for 12 h. Cell viability was measured using the CCK-8 assay (*n* = 6). **c** Cell viability measurement using the CCK-8 assay (*n* = 6). **d**, **e** MDA and GSH contents (*n* = 3). **f** Lipid-mediated ROS production detected by flow cytometry after staining SH-SY5Y cells with Bodipy (*n* = 3). **g** A heatmap of the relative levels of lipid metabolites in SH-SY5Y cells. Red indicates higher relative levels and blue depicts lower relative levels (*n* = 5). **h** Volcano plot of the LPLs. Colored points indicate individual LPC or LPE species, as defined in the figure key, whereas gray points indicate metabolites without significant differences. **i** Protein expressions of ALOX15 and GPX4 (*n* = 3). **j**, **k** MDA and GSH contents (*n* = 3). **l** Lipid-mediated ROS production among the SH-SY5Y cells transfected with either *ALOX15* overexpression plasmid or control plasmid detected by flow cytometry after staining with Bodipy. **m** SH-SY5Y cells were transfected with an overexpression or shRNA plasmid targeting *ALOX15*, followed by treatment with ferrostatin-1 (10 µM) or erastin (5 µM) for 24 h. Cell viability was measured using the CCK-8 assay Data are presented as the mean ± SD of at least three independent experiments. The differences among groups were analyzed using one-way ANOVA with Tukey’s post hoc test or Student’s t-test. Significance was determined at a *p*-level of * *p* < 0.05; ** *p* < 0.01; *** *p* < 0.001; ns = no significance

To further validate the role of ALOX15 in HYP-induced injury at the cellular level, we performed in vitro experiments using SH-SY5Y cells. First, a time-course analysis was conducted to determine an appropriate HYP exposure duration. HYP exposure for 8 h did not significantly affect cell viability or ferroptosis-related markers, including ALOX15 expression (Fig. S3a–c). In contrast, 12 h of HYP exposure markedly reduced cell viability and was accompanied by increased ALOX15 expression and ferroptosis-related changes (Fig. 5a and Fig. S3a–c). Therefore, 12 h of HYP exposure was selected for subsequent in vitro experiments. To determine whether the observed cell death was related to ferroptosis, cells were treated with different cell death inhibitors. Fer-1, a ferroptosis inhibitor that suppresses lipid peroxidation, rescued HYP-induced cell death, whereas necrostatin-1, a necroptosis inhibitor, and Z-VAD-FMK, a pan-caspase apoptosis inhibitor, did not show comparable protective effects (Fig. 5b). These results suggest that ferroptosis is involved in HYP-induced SH-SY5Y cell injury. To further strengthen the specificity of ferroptosis involvement, we performed additional pharmacological experiments. Deferasirox (DFX), an iron chelator, and Liproxstatin-1, a lipid ROS scavenger, both attenuated HYP-induced reductions in cell viability and alleviated MDA accumulation, GSH depletion, and lipid peroxidation (Fig. S4). We next examined whether *ALOX15* knockdown could attenuate this process. Genetic knockdown of *ALOX15* reduced HYP-induced cell death, restored GSH levels, and decreased the accumulation of MDA and lipid peroxides (Fig. 5c–f). Consistently, lipidomic profiling showed that HYP-induced depletion of LPC18:0 and elevation of PL products were partially reversed by ALOX15 inhibition (Fig. 5g, h).

To further assess the functional contribution of ALOX15, gain-of-function experiments were performed under normoxia. *ALOX15* overexpression decreased GPX4 and GSH levels while increasing MDA accumulation and lipid peroxidation, suggesting that *ALOX15* upregulation may be sufficient to promote ferroptosis-like changes (Fig. 5i–l). In addition, *ALOX15* knockdown reduced erastin-induced ferroptotic injury, whereas *ALOX15* overexpression increased cellular sensitivity to HYP-mimetic injury, which could be partially rescued by Fer-1 treatment (Fig. 5m)**.** Moreover, *GPX4* overexpression rescued HYP- or *ALOX15* overexpression-induced ferroptosis-related changes, including reduced cell viability, increased MDA accumulation, GSH depletion, and lipid peroxidation, providing additional genetic evidence for ferroptosis involvement (Fig. S5a-g). Together, these in vitro findings indicate that ALOX15 contributes to HYP-induced SH-SY5Y cells ferroptosis, possibly through PL/LPL metabolic disturbance and impaired antioxidant defense.

ALOX15 is constitutively expressed in multiple brain cell types, including microglia, neurons, and endothelial cells [18]. Given the critical role of intercellular communication in brain homeostasis, and considering that microglia serve as primary responders to hypoxic/ischemic insults [23], we explored whether microglial ALOX15 inhibition could influence neuronal ALOX15 expression through soluble factors. Neurons were treated with conditioned medium (CM) from BV2 cells transfected with sh*ALOX15* or shNEG and then exposed to HYP for 12 h (Fig. S6a). CM from shNEG-treated microglia did not markedly alter neuronal ALOX15 expression, whereas CM from sh*ALOX15*-treated microglia reduced neuronal ALOX15 levels (Fig. S6b). Transcript analysis showed reduced *Il6* and *Il1β* and increased Il10 in sh*ALOX15*-treated microglia (Fig. S6c). Exogenous IL-10 administration reduced ALOX15 expression in hypoxic SH-SY5Y cells, suggesting that IL-10 may be involved in the conditioned-medium-mediated effect on neuronal ALOX15 expression in vitro (Fig. S6d). However, because these findings are mainly based on CM experiments, they should be interpreted as supportive evidence for possible microglia–neuron communication rather than definitive proof of an in vivo microglia–neuron regulatory axis.

### Arachidonate 15-lipoxygenase-regulated hypoxia injury is associated with phospholipase A2 group IVC suppression

**Fig. 6:**
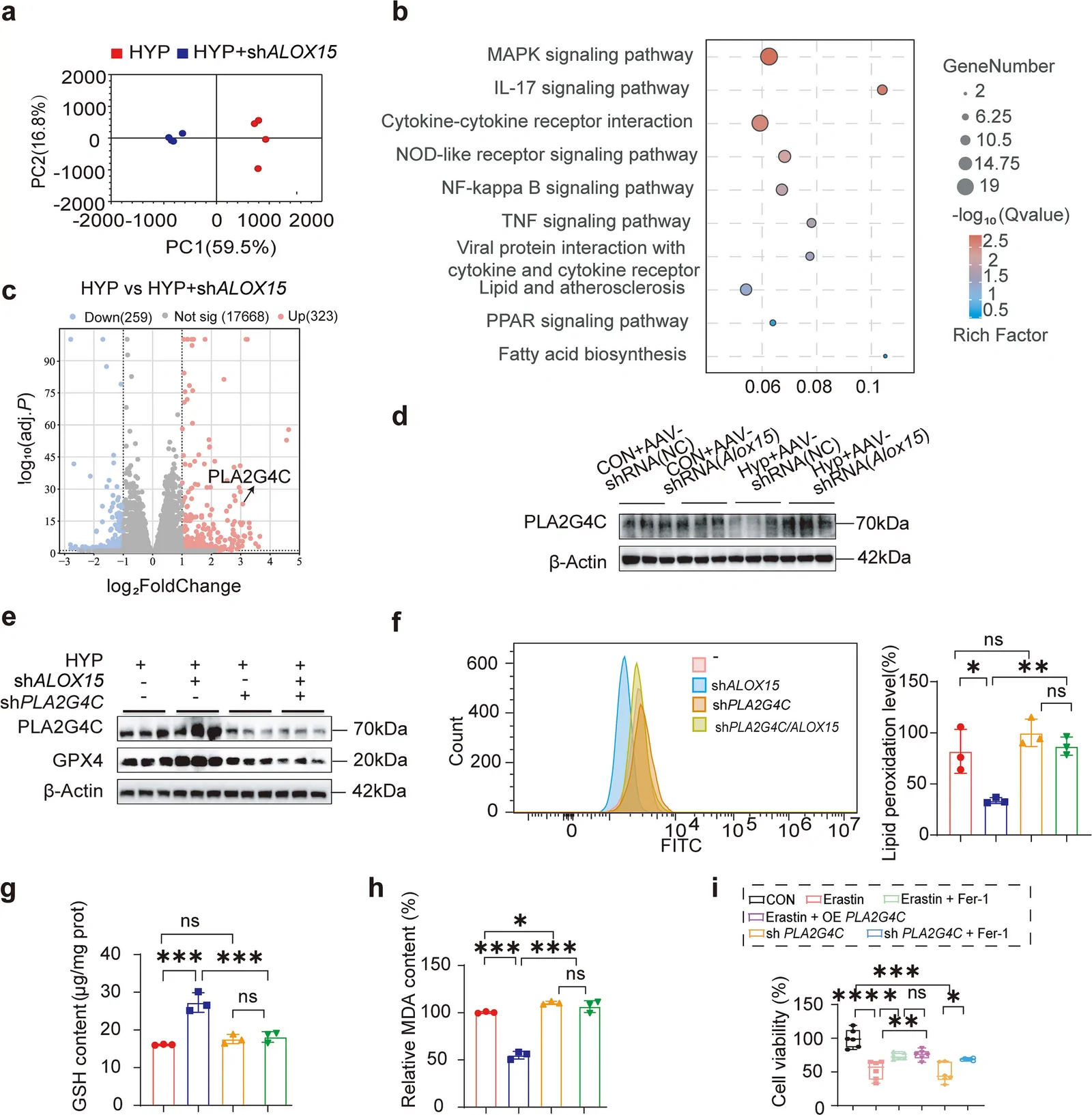
Arachidonate 15-lipoxygenase exacerbates hypoxia-induced neuronal injury by suppressing phospholipase A2 group IVC expression and reducing LPC18:0 levels. **a** PCA analysis of gene pattern changes in the SH-SY5Y cells, each point represents one independent biological sample (*n* = 4). **b** Kyoto Encyclopedia of Genes and Genomes (KEGG) pathways associated with the DEGs. Bubble size indicates the number of genes enriched in each pathway. **c** A volcano plot of the differences in gene expressions. Red points indicate significantly upregulated genes, blue points indicate significantly downregulated genes, and gray points indicate genes without significant changes. **d** Representative Western blot images and statistical data of PLA2G4C protein expression in hippocampal tissues of mice (*n* = 3). **e** Representative Western blot images of the PLA2G4C and GPX4 protein expressions (*n* = 3). **f** Lipid-mediated ROS production in SH-SY5Y cells detected by flow cytometry after staining with Bodipy (*n* = 3). **g**, **h** Relative intracellular MDA content in hypoxia-treated SH-SY5Y cells transfected with control shRNA, sh*ALOX15*, sh*PLA2G4C*, or combined sh*PLA2G4C* and sh*ALOX15*. Values were normalized to the control group (*n* = 3). **i** SH-SY5Y cells were transfected with either *PLA2G4C* overexpression plasmid or *PLA2G4C*-targeting shRNA plasmid, followed by 24-h treatment with erastin (5 μM) or ferrostatin-1 (10 μM), respectively. Cell viability was measured using the CCK-8 assay (*n* = 6). The differences among groups were analyzed using one-way ANOVA with Tukey’s post hoc test. Significance was determined at a *p*-level of * *p* < 0.05; ** *p* < 0.01; *** *p* < 0.001; ns = no significance

To explore the dow2nstream mechanism of ALOX15 in HYP injury, RNA-seq was performed in HYP-treated cells with or without *ALOX15* knockdown. PCA showed clear separation between the HYP and ALOX15-inhibited HYP groups (Fig. 6a). Pathway enrichment analysis identified MAPK signaling, inflammation, and lipid metabolism as major altered pathways (Fig. 6b). Phospholipase A2 group IVC (PLA2G4C), a cytosolic phospholipase A2 (cPLA2), was among the most upregulated genes within the MAPK signaling pathway (Fig. 6c). Validation experiments further showed that *ALOX15* knockdown increased PLA2G4C expression in mouse hippocampal tissues and cells, whereas *ALOX15* overexpression reduced PLA2G4C expression (Fig. 6d and Fig. S7a, b). In contrast, *PLA2G4C* knockdown or overexpression did not alter ALOX15 expression (Fig. S7c, d). Functionally, *PLA2G4C* overexpression attenuated HYP-induced ferroptosis (Fig. S7e).

To determine whether PLA2G4C mediates the downstream effects of *ALOX15, PLA2G4C* was overexpressed under *ALOX15*-overexpressing conditions during HYP exposure. *PLA2G4C* overexpression partially reversed *ALOX15* overexpression-induced ferroptosis-related changes, including GPX4 reduction, GSH depletion, and MDA accumulation (Fig. S8a–f). Conversely, *PLA2G4C* knockdown weakened the protective effect of ALOX15 inhibition against HYP-induced ferroptosis, as shown by altered GPX4, GSH, MDA, and lipid peroxidation levels (Fig. 6e–h). Fer-1 partially rescued *PLA2G4C* knockdown-induced cell death, and *PLA2G4C* overexpression reduced erastin-induced ferroptotic cell death (Fig. 6i). These findings suggest that PLA2G4C functions as a potential downstream effector of ALOX15 in regulating HYP-induced ferroptosis.

To further explore how ALOX15 regulates PLA2G4C expression, we analyzed potential upstream transcriptional regulators of PLA2G4C using RNA-seq data combined with JASPAR database prediction. MAFK and CREM were identified as candidate transcription factors, and their involvement was supported by RT-qPCR validation and siRNA-mediated knockdown experiments (Fig. S9a-f). Because MAFK showed a more dynamic response to HYP-associated ALOX15 regulation, we further examined its role in PLA2G4C transcription. *MAFK* overexpression enhanced PLA2G4C promoter activity, and promoter truncation analysis indicated that the −1000 to −500 bp region was important for MAFK-dependent activation (Fig. S9g, h). Moreover, *ALOX15* overexpression attenuated MAFK-induced PLA2G4C expression and suppressed MAFK-mediated PLA2G4C promoter activation (Fig. S9i, j). These findings suggest that MAFK may serve as an important transcriptional mediator linking ALOX15 regulation to PLA2G4C expression.

### Phospholipase A2 group IVC-derived LPC18:0 reverses the ferroptosis induced by hypoxia or phospholipase A2 group IVC suppression

**Fig. 7:**
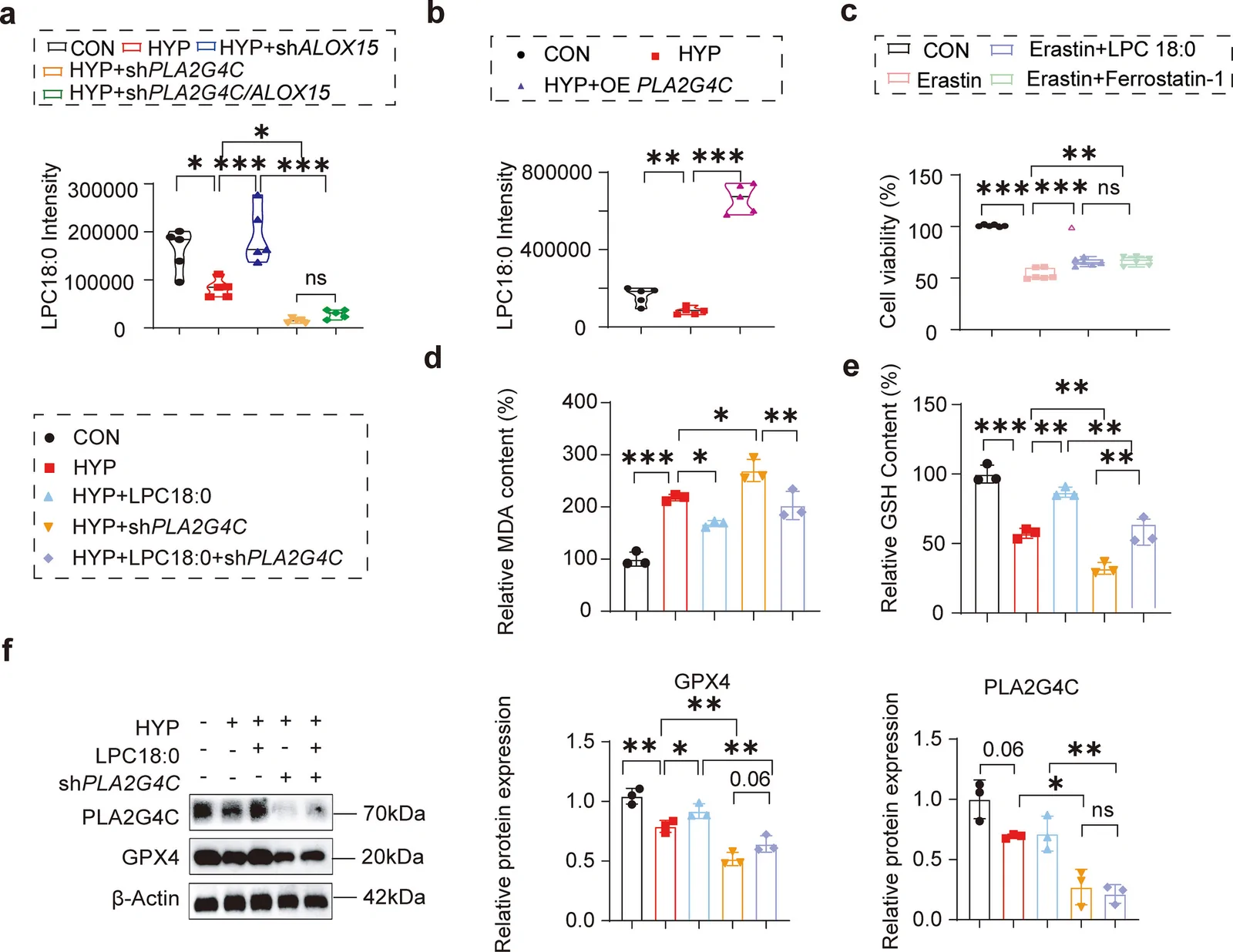
LPC18:0 alleviates hypoxia-induced ferroptosis in SH-SY5Y cells. **a**, **b** A violin plot of LPC18:0 expression in SH-SY5Y cells detected by a targeted MS assay (*n* = 5). **c** A violin plot of LPC18:0 expression in SH-SY5Y cells with *PLA2G4C* overexpression detected by a targeted MS assay (*n* = 5). **d** SH-SY5Y cells were pretreated with LPC18:0 (500 nM) for 20 h followed by incubation with erastin (5 µM) for 24 h. Cell viability was measured using the CCK-8 assay (*n* = 6). **e**, **f** Relative MDA and GSH contents in CON, HYP, HYP + LPC18:0, HYP + sh*PLA2G4C*, and HYP + LPC18:0 + sh*PLA2G4C* cells. LPC18:0 was administered at 500 nM for 20 h before HYP exposure. Values were normalized to those of the CON group. **g** Protein expression of GPX4 in SH-SY5Y cells (*n* = 3). Data are presented as the mean ± SD of at least three independent experiments. The differences among groups were analyzed using one-way ANOVA with Tukey’s post hoc test or Student’s t-test. Significance was determined at a *p*-level of * *p <* 0.05; ** *p <* 0.01; ***p < 0.001; ns = no significance

LPC species are generated from PC through PLA2-mediated hydrolysis and function as bioactive lipid mediators. Because the ALOX15–PLA2G4C pathway modulated LPC18:0 levels under HYP (Fig. 7a), we examined whether PLA2G4C affects LPC18:0 generation. PLA2G4C overexpression partially restored HYP-reduced LPC18:0 levels (Fig. 7b). LPC18:0 or Fer-1 treatment also reduced erastin-induced ferroptotic cell death (Fig. 7c), and LPC18:0 supplementation attenuated shPLA2G4C-enhanced ferroptosis-related changes under HYP, including altered MDA, GSH, and GPX4 levels (Fig. 7d–f). These data suggest that reduced PLA2G4C-derived LPC18:0 may contribute to ferroptosis under HYP.

### Systemic LPC18:0 supplementation alleviates hypoxia-induced cognitive impairment through suppression of hippocampal ferroptosis

Emerging evidence links plasma-derived LPCs to neurodevelopment and cognitive maintenance, and LPC depletion has been reported in AD patients [24]. Similarly, HYP-induced CI mice showed reduced LPC18:0 levels compared with normoxic controls. To preliminarily evaluate the systemic exposure and brain-associated distribution of LPC18:0, mice received a single intraperitoneal injection of LPC18:0 at 20 mg/kg. Serum LPC18:0 concentrations increased rapidly and reached a maximum concentration (Cmax) of approximately 4.2 × 10^3^ ng/mL at 2 h (Tmax). Preliminary non-compartmental analysis yielded an estimated AUC0–24 h of approximately 3.4 × 10^4^ ng·h/mL. Serum concentrations subsequently declined but remained detectable at 24 h. Hippocampal LPC18:0 levels increased more gradually, reaching a peak at approximately 6 h before declining, indicating delayed brain-associated distribution relative to systemic exposure (Fig. S10a, b). LPC18:0 transport was further evaluated using a bEnd.3 cell-based Transwell BBB model. LPC18:0 concentrations in the lower chamber increased to approximately 249 ng/mL at 1 h and remained relatively stable through 4 h (Fig. S10c, d).

**Fig. 8:**
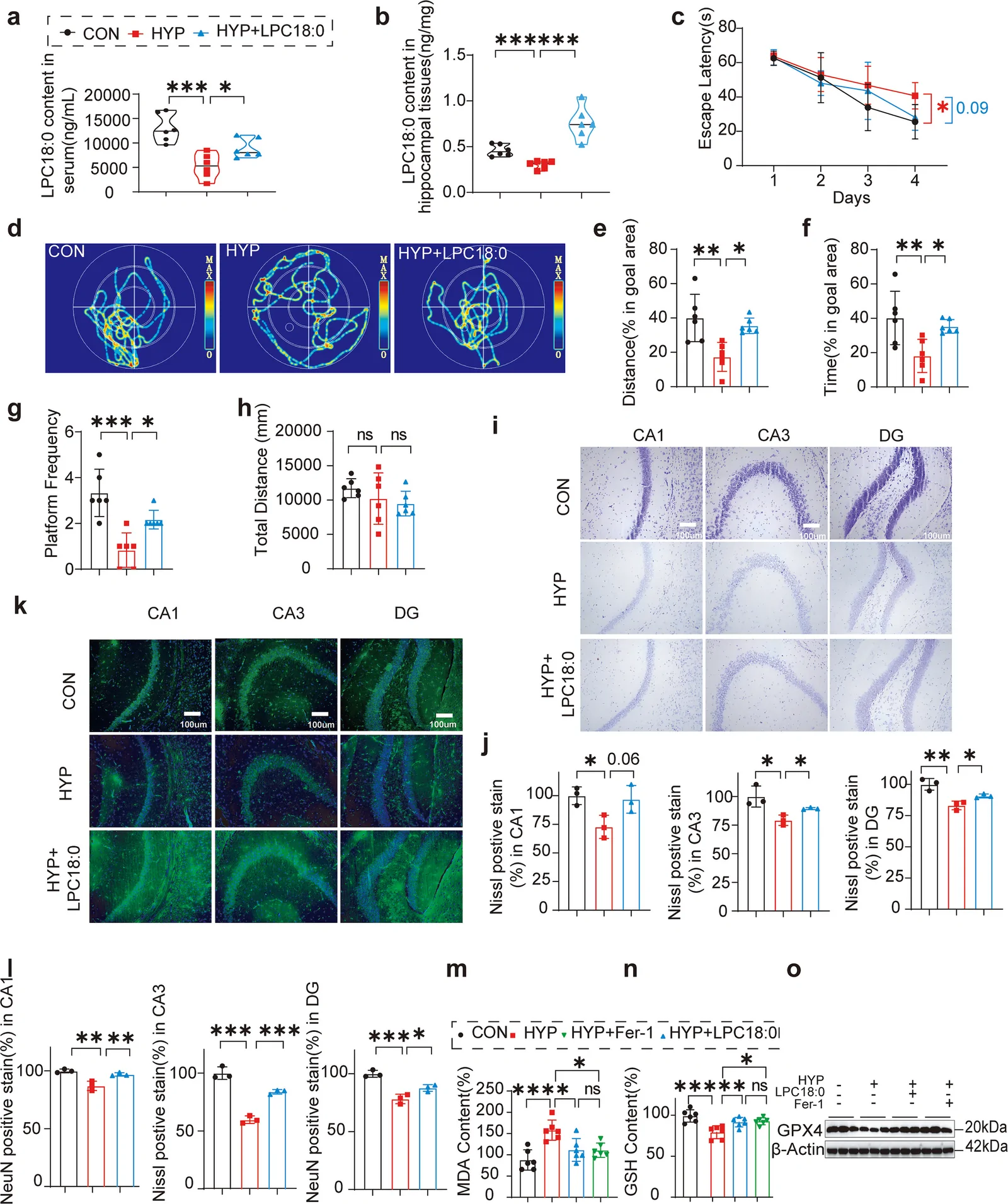
Supplementation of LPC18:0 alleviates hypoxia-induced cognitive impairment by suppressing neuronal ferroptosis. **a** A violin plot of the LPC18:0 contents in the serum assessed by the targeted MS assay. **b** The content of LPC18:0 in the hippocampal tissues (*n* = 6). **c** The escape latency of mice during the positioning navigation test. **d** The trajectories, (**e**) The percentages of the distance, (**f**) the time in the goal area, (**g**) the number of crossings over the original platform location, and (**h**) the total distance of exploration of mice in the spatial exploration swimming test (*n* = 6). **i** Representative images of Nissl body staining. Nissl-positive neuronal cell bodies are stained blue-purple. Scale bar, 100 μm. **j** Quantitative results of the Nissl staining in the hippocampal CA1, CA3, and DG regions of mice. **k** Representative images of NeuN staining. NeuN-positive neurons are shown in green, and nuclei counterstained with DAPI are shown in blue. White boxes in the whole-hippocampus images indicate the regions displayed at higher magnification. Scale bars, 100 μm. **l** Quantitative results of the NeuN staining in the hippocampal CA1, CA3, and DG regions of mice (*n* = 3). **m**, **n** Hippocampal MDA and GSH levels in CON mice and HYP mice treated with vehicle, ferrostatin-1 (Fer-1), or LPC18:0. MDA and GSH contents were normalized to the CON group (*n* = 6). **o** Representative Western blot images of GPX4 protein expression in the hippocampal tissues of mice (*n* = 3). Data are presented as the mean ± SD of at least three independent experiments. The differences among groups were analyzed using one-way ANOVA with Tukey’s post hoc test. Significance was determined at a *p*-level of * *p* < 0.05; ** *p* < 0.01; *** *p* < 0.001; ns = no significance

To investigate the therapeutic relevance of LPC restoration, HYP mice were intraperitoneally administered LPC18:0 for 21 days and subjected to behavioral testing. LC–MS/MS showed increased LPC18:0 concentrations in serum and hippocampal tissues after administration, suggesting systemic exposure and brain-associated distribution (Fig. 8a, b). Behavioral testing showed that LPC18:0 reduced escape latency, improved probe trial performance, increased target quadrant preference, and enhanced platform crossing frequency without affecting total swimming distance (Fig. 8c–h). Histological analysis further showed increased Nissl body density and NeuN staining in the hippocampus after LPC18:0 treatment (Fig. 8i–l). These findings suggest that LPC18:0 supplementation alleviates HYP-induced CI in mice. To further evaluate systemic lipid metabolic effects of LPC18:0 supplementation, serum PL/LPL profiles were analyzed. Heatmap analysis showed that HYP disrupted circulating LPC/LPE and PC/PE profiles, whereas LPC18:0 treatment partially normalized these lipid alterations (Fig. S11a–c). We next examined whether the protective effect of LPC18:0 was associated with ferroptosis suppression. Similar to Fer-1, LPC18:0 treatment decreased MDA levels and increased GSH and GPX4 levels in HYP mice (Fig. 8m–o). These findings suggest that LPC18:0 may act as an endogenous lipid mediator involved in ferroptosis regulation during HYP-induced brain injury.

## Discussion

Demographic shifts have increased the number of individuals exposed to high-altitude or hypoxic environments, making HYP-associated neurological dysfunction an important public health concern [25]. Consistent with previous evidence linking HYP to neurological dysfunction [12], our study found that HYP exposure impaired spatial learning and memory and induced hippocampal injury in mice. Mechanistic studies have indicated that ferroptosis contributes to ROS generation, membrane destabilization, and lipid peroxidation under severe HYP [26, 27], which is also implicated in neurodegeneration [28–30]. However, most prior studies have mainly described associations among HYP, oxidative stress, and neuronal damage, whereas the lipid metabolic mechanism connecting HYP to hippocampal ferroptosis and CI remains less defined. Our findings extend these observations by linking HYP-induced cognitive dysfunction to ferroptosis-associated PL/LPL remodeling in the hippocampus.

A major finding of this study is that ALOX15 may participate in HYP-induced hippocampal ferroptosis. Previous studies have mainly investigated ALOX15 in cancer-related ferroptosis, inflammatory disorders, or classical neurodegenerative contexts, whereas its role in HYP-induced hippocampal injury and cognitive dysfunction has been less clearly defined [31–33]. In the present study, ALOX15 inhibition attenuated ferroptosis-related biochemical changes and partially restored PL/LPL homeostasis, suggesting that ALOX15 may act as an upstream regulator of HYP-associated lipid peroxidation and lipid remodeling in hippocampal neurons. Lipid homeostasis depends on coordinated pathways regulating fatty acid/PL synthesis (ELOVL5 and FADS1), oxidation (ALOX15 and CYP450), and metabolism or clearance (PLA2G6, GPX4) [34–39]. Previous studies have highlighted that perturbations in these pathways under hypoxic or neurodegenerative conditions can predispose neurons to ferroptosis [40]. In line with this, HYP disrupted hippocampal PL/LPL balance and increased lipid peroxidation, resembling ferroptotic signatures reported in AD and ischemic stroke models. Silencing Alox15 modulated both PLA2G4C and GPX4 expression, suggesting that ALOX15 may influence ferroptotic susceptibility through lipid remodeling and antioxidant defense.

Our lipidomic results further link HYP-induced ferroptosis with membrane lipid remodeling. Previous redox lipidomics studies have shown that oxidized arachidonoyl- and adrenoyl-containing phosphatidylethanolamines are important ferroptotic lipid signals. Consistently, we observed marked changes in PE species, including PE-18:0/C20:4 and PE-18:0/C22:4, under HYP conditions. This supports the idea that HYP may promote ferroptosis through remodeling of peroxidation-prone PL species. At the same time, our study differs from previous ferroptosis studies by emphasizing the reduction of LPL species, particularly LPCs and LPEs. These findings suggest that ferroptosis under HYP may involve not only accumulation of oxidized PLs, but also impaired conversion or remodeling of PLs into LPL metabolites [41–43].

The PLA2 superfamily catalyzes hydrolysis of sn-2 acyl bonds in PLs and includes secretory (sPLA2), calcium-independent (iPLA2), lipoprotein-associated (Lp-PLA2), and cytosolic (cPLA2) subtypes [44–46]. Previous studies showed that iPLA2β suppresses ferroptosis by hydrolyzing oxidized phosphatidylethanolamine containing 15-hydroperoxyeicosatetraenoic acid [47, 48], while Lp-PLA2 also exhibits anti-ferroptotic effects through lipid metabolic remodeling [49, 50]. In contrast, cPLA2 may promote lipid peroxidation via AA liberation [51], highlighting the functional dichotomy within this enzyme family. Our data suggest that ALOX15 inhibition is associated with increased PLA2G4C expression and partial restoration of PL/LPL remodeling under HYP conditions. Because PLA2 enzymes can hydrolyze PLs or oxidized PLs to generate LPLs, PLA2G4C may contribute to the restoration of PL/LPL balance and the production of LPL metabolites such as LPC18:0. Conversely, PLA2G4C knockdown weakened the protective effect of ALOX15 inhibition, supporting PLA2G4C as a potential downstream effector in ALOX15-associated PL/LPL dysregulation and ferroptosis-related responses. Based on these findings, the PLA2G4C-mediated regulation of ferroptosis can be described in a stepwise manner. First, HYP-induced ALOX15 upregulation enhances PL peroxidation and disrupts PL/LPL homeostasis. Second, reduced PLA2G4C expression may limit the hydrolysis of PLs or oxidized PLs, thereby impairing the remodeling of PL/LPL balance and decreasing the generation of LPL metabolites. Third, LPL deficiency weakens the hippocampal capacity to buffer ferroptotic lipid stress, leading to increased lipid peroxidation, GPX4 depletion, GSH reduction, and MDA accumulation. Finally, restoration of PLA2G4C expression partially restores PL/LPL balance and suppresses ferroptotic injury. In future studies, it will be important to clarify the contribution of each PLA2 subtype to hippocampal lipid remodeling and ferroptosis-related injury under HYP.

Although PLA2 enzymes regulate PE, phosphatidylinositol, phosphatidylserine, and LPL levels [47, 50, 52], the precise PLA2 subtype involved in ferroptosis regulation under HYP remains unclear. Specific LPC species, such as LPC16:0 and LPC18:1, have been reported to inhibit RSL3-induced ferroptosis [50]. In this study, PLA2G4C increased LPL production, particularly LPC18:0, a bioactive lipid species associated with neuroprotection and ferroptosis inhibition [24, 34]. This interpretation is consistent with recent evidence suggesting that certain LPC species may reach the brain and modulate ferroptosis-related pathology through the GPR119–ACSL4 pathway. Similarly, our data suggest that LPC18:0 supplementation restores PL/LPL homeostasis and limits lipid peroxidation. Compared with studies examining ALOX15, PLA2 subtypes, or LPC metabolism separately, our study integrates these components into an ALOX15–PLA2G4C–LPC18:0 axis. This integrated mechanism may help explain how HYP-induced lipid peroxidation is coupled with impaired lipid remodeling and injury in the hippocampus. Our findings suggest that modulating this axis through ALOX15 inhibition, PLA2G4C restoration, or LPC18:0 supplementation may provide potential directions for protecting cognitive function under hypoxic conditions.

This study has several limitations. First, SH-SY5Y and BV2 cells cannot fully recapitulate primary neuronal and microglial biology, and the in vivo relevance of microglia-neuron communication and IL-10/IL-10R intervention requires validation. Second, the cell-specific role of ALOX15 requires further confirmation using neuron-specific Alox15 knockout models. Finally, clinical validation and tracer-labeled LPC18:0 distribution or BBB transport studies are needed to confirm translational relevance, delivery, and uptake mechanisms.

## Conclusion

This study suggests that the ALOX15–PLA2G4C–LPC18:0 axis may contribute to HYP-induced CI by regulating hippocampal lipid remodeling and ferroptosis-related injury. HYP was associated with ALOX15 upregulation, PL/LPL metabolic disturbance, LPC18:0 deficiency, lipid peroxidation, and ferroptosis-related damage. ALOX15 inhibition alleviated injury and partially restored lipid metabolic balance, while PLA2G4C appeared to act as a downstream effector involved in ALOX15-regulated LPC18:0 homeostasis. Moreover, LPC18:0 supplementation reduced ferroptosis-associated damage and improved cognitive performance in HYP-exposed mice. Overall, these findings provide insight into lipid metabolic regulation of ferroptosis under HYP and suggest that targeting ALOX15 or restoring LPC18:0 homeostasis may be a potential strategy for mitigating HYP-induced CI.

## Materials and methods

### Animals and experimental grouping

Six-week-old male C57BL/6 mice (22–24 g) and Sprague Dawley rats (190–200 g) were purchased from Zhejiang Weitong Lihua Laboratory Animal Technology Co., Ltd. Only male animals were used to minimize potential confounding effects of estrogen-related fluctuations in lipid metabolism and behavior, as supported by previous literature [53]. C57BL/6 mice were used for the main in vivo experiments, including the HYP-induced CI model, behavioral testing, AAV-mediated *Alox15* knockdown, LPC18:0 or Fer-1 treatment, histological analysis, biochemical assays, and lipidomic validation, whereas Sprague Dawley rats were used to validate selected ferroptosis-related candidate genes from public HYP-associated microarray data.

All animal experiments were reported in accordance with the ARRIVE guidelines. Animals were housed in a specific pathogen-free facility under controlled environmental conditions at 22 ± 2 °C and 50%–60% relative humidity, with a 12-h light/12-h dark cycle and lights on at 08:00. Standard laboratory chow and water were provided ad libitum. Mice were acclimatized for 2 weeks and randomly assigned to experimental groups. For the primary HYP-induced CI experiment, mice were divided into control and HYP groups (*n* = 8 per group; Fig. S12a). For AAV-mediated Alox15 knockdown, mice were assigned to AAV-shNC, AAV-sh*Alox15*, HYP + AAV-shNC, and HYP + AAV-shAlox15 groups (*n* = 8 per group; Fig. S12b). For LPC18:0 and Fer-1 intervention experiments, mice were assigned to control, HYP, and HYP + LPC18:0 or Fer-1 groups (*n* = 6 per group; Fig. S12c). For rat-based validation, rats were acclimatized for 1 week and assigned to control and HYP groups (*n* = 3 per group; Fig. S12d). Exact animal numbers for each assay are provided in the corresponding figure legends.

### Hypoxia model establishment

The HYP model was established using a high-low pressure oxygen chamber (Tow-int tech, Shanghai, China, ProOx-850). The chamber was set to simulate an altitude of 7620 m, corresponding to approximately 38.6% of normal atmospheric pressure and oxygen partial pressure, with an effective oxygen content of approximately 8% O₂. Animals were continuously exposed to this hypobaric hypoxic condition for 7 consecutive days under controlled alternating light/dark cycles. The exposure was continuous, and oxygen level was maintained throughout the experiment.

### Bioinformatic screening of ferroptosis-related candidate genes

The HYP-associated microarray dataset was obtained from the NCBI GEO database by searching the terms “hypobaric hypoxia” and “hippocampus.” The GSE66287 dataset was selected because it included hippocampal samples from rats exposed to HYP for 1, 3, or 7 days, with five animals in each group. Raw microarray data were processed in R software (version 4.2.1) using the limma package (version 3.52.4), and normalization was performed with the “normalize Between Arrays” function [54]. DEGs were screened using the thresholds |log2FC|> 1 and adjusted *p*-value < 0.05, and volcano plots were generated using the ggplot2 package. FRGs were obtained from the FerrDb v2 database, which contains 433 ferroptosis drivers, suppressors, and markers. Candidate ferroptosis-related DEGs were identified by intersecting DEGs with FRGs and visualized using Venn diagrams. The expression profiles of overlapping genes were displayed using the pheatmap package (version 1.0.12). A PPI network was then constructed, and the top five central node genes were identified using the Molecular Complex Detection (MCODE) plugin in Cytoscape software (version 3.9.1). Correlation analysis of these central node genes was further performed in R to evaluate their relationships. The overall bioinformatic workflow is shown in Fig. S12e.

### Quantitative real-time polymerase chain reaction analysis

Total RNA was extracted from hippocampal tissues and cultured cells using TRIzol reagent (Takara, Shiga-ken, Japan) according to the manufacturer’s instructions. The extracted RNA was dissolved in RNase-free water, and its concentration and purity were determined using a NanoDrop spectrophotometer (Thermo Fisher Scientific, USA). Equal amounts of RNA were reverse-transcribed into cDNA using HiScript III RT SuperMix for qPCR (+ gDNA wiper) (Vazyme Biotech Co., Ltd., Nanjing, China).

qRT-PCR was performed using ChamQ Universal SYBR qPCR Master Mix (Vazyme Biotech Co., Ltd.) on a QuantStudio™ 5 Real-Time PCR System (Applied Biosystems, USA). The reactions were conducted in a 20 μL volume containing SYBR Green Master Mix, gene-specific primers, cDNA template, and nuclease-free water. A melting curve analysis was performed to verify amplification specificity. Relative gene expression levels were calculated using the 2^-ΔΔCt method, with GAPDH as the endogenous reference control. All reactions were performed with technical replicates, and primer sequences are provided in Table S1.

### AAV-mediated arachidonate 15-lipoxygenase knockdown in the hippocampus

AAV vectors targeting Alox15 or the negative control were generated by Shanghai OBiO Technology Co., Ltd. The viral serotype was AAV9, with a titer of 1.0E + 12 vg/mL. The vectors were pAAV-U6-shRNA(Alox15)-CMV-EGFP-WPRE and pAAV-U6-shRNA(NC)-CMV-EGFP-WPRE. The AAV-shAlox15 target sequence was CACTCTGTTTGAAGCGGATTT, and the scrambled control sequence was TTCTCCGAACGTGTCACGT. AAV vectors were bilaterally injected into the hippocampus at AP −2.0 mm, ML ±1.5 mm, and DV −2.0 mm. A total of 1 μL virus was injected at 50 nL/min, and the needle was retained for 10 min to prevent backflow. HYP exposure and behavioral/molecular analyses were performed 4 weeks after AAV injection. EGFP fluorescence was used to assess viral transduction distribution in the hippocampus.

### Behavioral tests

Spatial learning and memory were evaluated using the MWM test as previously described [22]. Mice were randomly assigned to experimental groups, and behavioral testing and data analysis were performed by investigators blinded to group allocation. Sample size was determined based on preliminary data and previous studies using similar HYP-induced CI models. During the 4-day training phase, mice were tested from different starting directions. If a mouse located the hidden platform within 65 s, it remained on the platform for 15 s; otherwise, it was guided to the platform and kept there for 15 s. On day 5, the platform was removed, and each mouse underwent a 65-s probe trial from the quadrant opposite the former platform location. Trajectories were recorded using an overhead camera and analyzed with MWT-100 Morris software (Taimeng Software Co., Ltd., Chengdu, China). Animals were excluded only according to predefined criteria, including death before test completion, severe unrelated illness or injury, motor impairment affecting swimming, or video-tracking failure.

### LC–MS-based lipidomic analysis of LPC18:0 and phospholipid/lysophospholipid metabolism

Lipids were extracted from cell pellets or hippocampal tissues. Briefly, approximately 20 mg of hippocampal tissue or an equivalent amount of harvested cell pellets was placed in a precooled microcentrifuge tube, and 400 μL of ice-cold isopropanol (IPA) containing the internal standard was added. Tissue samples were homogenized on ice, whereas cell pellets were thoroughly vortexed until completely dispersed. The samples were then incubated at 4 °C for 10 min to facilitate lipid extraction and protein precipitation, followed by centrifugation at 15,000 × g for 15 min at 4 °C. The resulting supernatant was carefully transferred to a clean tube, dried under a gentle stream of nitrogen, and reconstituted in 100 μL IPA. After vortexing for 2 min and centrifugation at 15,000 × g for 15 min, the clarified supernatant was transferred to autosampler vials for LC–MS/MS analysis.

Lipidomic profiling was performed using a SHIMADZU CBM-30A Lite LC system coupled to an API 6500 Q-TRAP mass spectrometer (AB SCIEX, Foster City, CA, USA) in both ESI-positive and ESI-negative modes. Separation was achieved on a Phenomenex Kinetex C18 column (2.1 × 100 mm, 2.6 μm) at 40 °C with an injection volume of 1 μL. The mobile phases were (A) H₂O/MeOH/ACN (3:1:1, v/v/v) containing 5 mM ammonium formate and (B) IPA. The gradient was as follows: 25% B for 0–0.5 min, 25%–40% B for 0.5–1.5 min, 40%–60% B for 1.5–3 min, 60%–98% B for 3–13 min, 98%–25% B for 13–13.1 min, and 25% B for 13.1–18 min. PLs and LPLs were quantified by multiple reaction monitoring (MRM) using PE18:1 (200 ng/mL; GlpBio, USA) as the internal standard. To minimize potential analytical bias, sample preparation and injection order were randomized. A pooled quality-control sample was prepared by combining equal aliquots from all study samples and was injected at the beginning of the sequence and at regular intervals during analysis. Solvent blanks were also analyzed to monitor carryover and background contamination. Analytical stability was evaluated based on the retention times and peak areas of the internal standard and representative endogenous lipids. No evident retention-time drift or abnormal fluctuation in the internal-standard peak area was observed during the analytical sequence.

Raw MRM data were processed using AB SCIEX MultiQuant software version 2.1. Peak integration was manually inspected when necessary, and metabolite concentrations were calculated from the analyte-to-internal-standard peak-area ratios. Relative PL and LPL abundances were normalized to the internal standard and, where appropriate, to tissue weight, cell number, or total protein content. For absolute LPC18:0 quantification, external calibration standards ranging from 2 to 500 ng/mL were prepared. Calibration curves were constructed by plotting the LPC18:0-to-internal-standard peak-area ratio against the corresponding LPC18:0 concentration using [weighted/unweighted] linear regression. The calibration curve showed acceptable linearity over the tested range. The limits of detection and quantification were 1 and 2 ng/mL, respectively. The full MRM transition information is provided in Table S2.

### Cell culture and in vitro Hypoxia model for mechanistic validation

SH-SY5Y cells were maintained in DMEM/F12 medium (Biosharp, China) containing 10% fetal bovine serum (FBS; Gemini, Mexico) and 1% penicillin–streptomycin (Solarbio, China) at 37 °C in a humidified incubator with 5% CO₂. Cells were seeded into 6-well plates (2 × 10^5^ cells/well) or 96-well plates (2 × 10^3^ cells/well) and cultured overnight before treatment. For HYP induction, cells were placed in a hypoxia incubator (Ox-121C-50, Tow-int Tech, Shanghai, China) under 1% O₂, 5% CO₂, and balanced N₂ at 37 °C for 12 h. Cell viability was assessed using the Cell Counting Kit-8 (CCK-8; GLPBIO Biotechnology), and absorbance was measured at 450 nm with a microplate reader. BV2 cells, an immortalized microglial cell line with several characteristics of primary microglia [55], were cultured in high-glucose DMEM supplemented with 10% FBS and 1% penicillin–streptomycin under standard culture conditions at 37 °C in a humidified incubator with 5% CO₂. The culture medium was replaced every 2–3 days. When cells reached approximately 70%–80% confluence, they were washed twice with PBS and detached using 0.25% trypsin–EDTA. Trypsinization was terminated by adding complete culture medium, after which the cells were collected by centrifugation, resuspended in fresh medium, and passaged at a ratio of 1:3 to 1:5. Cells were routinely examined for morphology and growth characteristics using an inverted microscope. All experiments were performed using cells below passage 15 to minimize passage-associated phenotypic drift. All cell lines were authenticated by short tandem repeat profiling before use.

### RNA sequencing for downstream pathway analysis

RNA sequencing was performed using human SH-SY5Y neuroblastoma cells. Total RNA was extracted with TRIzol reagent (Sigma), and RNA quality was evaluated using the RNA Nano 6000 Assay Kit on a Bioanalyzer 2100 system (Agilent Technologies, CA, USA). cDNA libraries were prepared with the Fast RNA-seq Lib Prep Kit V2 (Cat. No. RK20306) and sequenced on the NovaSeq X Plus platform. After quality filtering, each RNA-seq sample generated 38.88–48.77 million clean reads, with an average of approximately 41.59 million clean reads per sample. After quality control, clean reads were mapped to the human reference genome GRCh38 using HISAT2 v2.0.5. Gene expression was calculated as FPKM using Cufflinks, and raw read counts were obtained with featureCounts v1.5.0-p3. DEGs were identified using DESeq2 (version 1.20.0) with p-value < 0.05 and fold change > 2 or < 0.5.

### Statistical analysis

Statistical analyses were performed using Prism software (GraphPad Prism version 8.00, USA), and continuous variables are expressed as the mean ± standard deviation (SD). Before applying parametric tests, data normality and homogeneity of variance were assessed using the Shapiro–Wilk test and Brown–Forsythe test, respectively. For datasets that satisfied the assumptions of normal distribution and equal variance, Student’s t-test was used for comparisons between two groups, and one-way analysis of variance (ANOVA) followed by Tukey’s post hoc test was used for multiple comparisons. For Morris water maze training data, escape latency was analyzed independently for each training day using one-way ANOVA followed by Tukey’s post hoc test, because the primary comparison focused on intergroup differences within the same training day rather than the overall longitudinal learning trajectory. Statistical significance was defined as * *p* < 0.05, ** *p* < 0.01, *** *p* < 0.001, ns, not significant.

## Supplementary Information

Supplementary Material 1.

## Data Availability

The metabolomics data are available in Synapse under Project SynID syn75980291. The transcriptome sequencing data are available in NCBI under accession number PRJNA1477317.

## References

[1] Hyder F, Rothman DL, Bennett MR. Cortical energy demands of signaling and nonsignaling components in brain are conserved across mammalian species and activity levels. Proc Natl Acad Sci U S A. 2013;110(9):3549–54. 10.1073/pnas.1214912110. 23319606 10.1073/pnas.1214912110 PMC3587194

[2] Burtscher J, Mallet RT, Burtscher M, Millet GP. Hypoxia and brain aging: Neurodegeneration or neuroprotection? Ageing Res Rev. 2021;68:101343. 10.1016/j.arr.2021.101343. 33862277 10.1016/j.arr.2021.101343

[3] West JB. High-altitude medicine. Am J Respir Crit Care Med. 2012;186(12):1229–37. 10.1164/rccm.201207-1323CI. 23103737 10.1164/rccm.201207-1323CI

[4] Su R, Jia S, Zhang N, Wang Y, Li H, Zhang D, et al. The effects of long-term high-altitude exposure on cognition: a meta-analysis. Neurosci Biobehav Rev. 2024;161:105682. 10.1016/j.neubiorev.2024.105682. 38642865 10.1016/j.neubiorev.2024.105682

[5] Herridge MS, Moss M, Hough CL, Hopkins RO, Rice TW, Bienvenu OJ, et al. Recovery and outcomes after the acute respiratory distress syndrome (ARDS) in patients and their family caregivers. Intensive Care Med. 2016;42(5):725–38. 10.1007/s00134-016-4321-8. 27025938 10.1007/s00134-016-4321-8

[6] Sasannejad C, Ely EW, Lahiri S. Long-term cognitive impairment after acute respiratory distress syndrome: a review of clinical impact and pathophysiological mechanisms. Crit Care. 2019;23(1):352. 10.1186/s13054-019-2626-z. 31718695 10.1186/s13054-019-2626-z PMC6852966

[7] Zhang M, Jiang WI, Arkelius K, Swanson RA, Ma DK, Singhal NS. PATJ regulates cell stress responses and vascular remodeling post-stroke. Redox Biol. 2025;85:103709. 10.1016/j.redox.2025.103709. 40472775 10.1016/j.redox.2025.103709 PMC12169792

[8] Li K, Luo R, Yu X, Dong W, Hao G, Hu D, et al. Enhanced human adipose-derived stem cells with VEGFA and bFGF mRNA promote stable vascular regeneration and improve cardiac function following myocardial infarction. Clin Transl Med. 2025;15(3):e70250. 10.1002/ctm2.70250. 40008489 10.1002/ctm2.70250 PMC11862888

[9] Mwaniki MK, Atieno M, Lawn JE, Newton CR. Long-term neurodevelopmental outcomes after intrauterine and neonatal insults: a systematic review. Lancet. 2012;379(9814):445–52. 10.1016/s0140-6736(11)61577-8. 22244654 10.1016/S0140-6736(11)61577-8 PMC3273721

[10] Inoue Y, Shue F, Bu G, Kanekiyo T. Pathophysiology and probable etiology of cerebral small vessel disease in vascular dementia and Alzheimer’s disease. Mol Neurodegener. 2023;18(1):46. 10.1186/s13024-023-00640-5. 37434208 10.1186/s13024-023-00640-5 PMC10334598

[11] Pocock R, Hobert O. Oxygen levels affect axon guidance and neuronal migration in Caenorhabditis elegans. Nat Neurosci. 2008;11(8):894–900. 10.1038/nn.2152. 18587389 10.1038/nn.2152

[12] Beinlich FRM, Asiminas A, Untiet V, Bojarowska Z, Plá V, Sigurdsson B, et al. Oxygen imaging of hypoxic pockets in the mouse cerebral cortex. Science. 2024;383(6690):1471–8. 10.1126/science.adn1011. 38547288 10.1126/science.adn1011 PMC11251491

[13] Li KC, Shi HX, Li Z, You P, Pan J, Cai YC, et al. Human DDIT4L intron retention contributes to cognitive impairment and amyloid plaque formation. Cell Discov. 2025;11(1):12. 10.1038/s41421-024-00759-9. 39929796 10.1038/s41421-024-00759-9 PMC11811001

[14] Zhang Y, Fang J, Dong Y, Ding H, Cheng Q, Liu H, et al. High-Altitude Hypoxia Exposure Induces Iron Overload and Ferroptosis in Adipose Tissue. Antioxidants (Basel). 2022;11(12):2367. 10.3390/antiox11122367. 36552575 10.3390/antiox11122367 PMC9774922

[15] Zhu X, Guan R, Zou Y, Li M, Chen J, Zhang J, et al. Cold-inducible RNA binding protein alleviates iron overload-induced neural ferroptosis under perinatal hypoxia insult. Cell Death Differ. 2024;31(4):524–39. 10.1038/s41418-024-01265-x. 38388728 10.1038/s41418-024-01265-x PMC11043449

[16] Conrad M, Kagan VE, Bayir H, Pagnussat GC, Head B, Traber MG, et al. Regulation of lipid peroxidation and ferroptosis in diverse species. Genes Dev. 2018;32(9–10):602–19. 10.1101/gad.314674.118. 29802123 10.1101/gad.314674.118 PMC6004068

[17] Dixon SJ, Lemberg KM, Lamprecht MR, Skouta R, Zaitsev EM, Gleason CE, et al. Ferroptosis: an iron-dependent form of nonapoptotic cell death. Cell. 2012;149(5):1060–72. 10.1016/j.cell.2012.03.042. 22632970 10.1016/j.cell.2012.03.042 PMC3367386

[18] Singh NK, Rao GN. Emerging role of 12/15-Lipoxygenase (ALOX15) in human pathologies. Prog Lipid Res. 2019;73:28–45. 10.1016/j.plipres.2018.11.001. 30472260 10.1016/j.plipres.2018.11.001 PMC6338518

[19] Cao Z, Liu X, Zhang W, Zhang K, Pan L, Zhu M, et al. Biomimetic Macrophage Membrane-Camouflaged Nanoparticles Induce Ferroptosis by Promoting Mitochondrial Damage in Glioblastoma. ACS Nano. 2023;17(23):23746–60. 10.1021/acsnano.3c07555. 37991252 10.1021/acsnano.3c07555 PMC10722604

[20] Liu F, Zuo X, Liu Y, Deguchi Y, Moussalli MJ, Chen W, et al. Suppression of Membranous LRP5 Recycling, Wnt/β-Catenin Signaling, and Colon Carcinogenesis by 15-LOX-1 Peroxidation of Linoleic Acid in PI3P. Cell Rep. 2020;32(7):108049. 10.1016/j.celrep.2020.108049. 32814052 10.1016/j.celrep.2020.108049 PMC8765570

[21] Li Y, Wang Y, Shi F, Zhang X, Zhang Y, Bi K, et al. Phospholipid metabolites of the gut microbiota promote hypoxia-induced intestinal injury via CD1d-dependent γδ T cells. Gut Microbes. 2022;14(1):2096994. 10.1080/19490976.2022.2096994. 35898110 10.1080/19490976.2022.2096994 PMC9336479

[22] Li Z, Fu J, Jiang K, Gao J, Guo Y, Li C, et al. Hyperbaric Oxygen Improves Cognitive Impairment Induced by Hypoxia via Upregulating the Expression of Oleic Acid and MBOAT2 of Mice. Antioxidants (Basel). 2024;13(11):1320. 10.3390/antiox13111320. 39594462 10.3390/antiox13111320 PMC11591255

[23] Kettenmann H, Hanisch UK, Noda M, Verkhratsky A. Physiology of microglia. Physiol Rev. 2011;91(2):461–553. 10.1152/physrev.00011.2010. 21527731 10.1152/physrev.00011.2010

[24] Zha X, Liu X, Wei M, Huang H, Cao J, Liu S, et al. Microbiota-derived lysophosphatidylcholine alleviates Alzheimer’s disease pathology via suppressing ferroptosis. Cell Metab. 2025;37(1):169-186.e9. 10.1016/j.cmet.2024.10.006. 39510074 10.1016/j.cmet.2024.10.006

[25] West JB. High-altitude medicine. Lancet Respir Med. 2015;3(1):12–3. 10.1016/s2213-2600(14)70238-3. 25466336 10.1016/S2213-2600(14)70238-3

[26] Zou Y, Palte MJ, Deik AA, Li H, Eaton JK, Wang W, et al. A GPX4-dependent cancer cell state underlies the clear-cell morphology and confers sensitivity to ferroptosis. Nat Commun. 2019;10(1):1617. 10.1038/s41467-019-09277-9. 30962421 10.1038/s41467-019-09277-9 PMC6453886

[27] Dixon SJ, Patel DN, Welsch M, Skouta R, Lee ED, Hayano M, et al. Pharmacological inhibition of cystine-glutamate exchange induces endoplasmic reticulum stress and ferroptosis. Elife. 2014;3:e02523. 10.7554/eLife.02523. 24844246 10.7554/eLife.02523 PMC4054777

[28] Yan HF, Zou T, Tuo QZ, Xu S, Li H, Belaidi AA, et al. Ferroptosis: mechanisms and links with diseases. Signal Transduct Target Ther. 2021;6(1):49. 10.1038/s41392-020-00428-9. 33536413 10.1038/s41392-020-00428-9 PMC7858612

[29] Stockwell BR, FriedmannAngeli JP, Bayir H, Bush AI, Conrad M, Dixon SJ, et al. Ferroptosis: A Regulated Cell Death Nexus Linking Metabolism, Redox Biology, and Disease. Cell. 2017;171(2):273–85. 10.1016/j.cell.2017.09.021. 28985560 10.1016/j.cell.2017.09.021 PMC5685180

[30] Moujalled D, Strasser A, Liddell JR. Molecular mechanisms of cell death in neurological diseases. Cell Death Differ. 2021;28(7):2029–44. 10.1038/s41418-021-00814-y. 34099897 10.1038/s41418-021-00814-y PMC8257776

[31] Jin G, Arai K, Murata Y, Wang S, Stins MF, Lo EH, et al. Protecting against cerebrovascular injury: contributions of 12/15-lipoxygenase to edema formation after transient focal ischemia. Stroke. 2008;39(9):2538–43. 10.1161/strokeaha.108.514927. 18635843 10.1161/STROKEAHA.108.514927 PMC2754072

[32] Joshi YB, Giannopoulos PF, Praticò D. The 12/15-lipoxygenase as an emerging therapeutic target for Alzheimer’s disease. Trends Pharmacol Sci. 2015;36(3):181–6. 10.1016/j.tips.2015.01.005. 25708815 10.1016/j.tips.2015.01.005 PMC4355395

[33] Yang H, Zhuo JM, Chu J, Chinnici C, Praticò D. Amelioration of the Alzheimer’s disease phenotype by absence of 12/15-lipoxygenase. Biol Psychiatry. 2010;68(10):922–9. 10.1016/j.biopsych.2010.04.010. 20570249 10.1016/j.biopsych.2010.04.010

[34] Karaki T, Haniu H, Matsuda Y, Tsukahara T. Lysophospholipids-potent candidates for brain food, protects neuronal cells against α-Synuclein aggregation. Biomed Pharmacother. 2022;156:113891. 10.1016/j.biopha.2022.113891. 36265307 10.1016/j.biopha.2022.113891

[35] Lee JY, Nam M, Son HY, Hyun K, Jang SY, Kim JW, et al. Polyunsaturated fatty acid biosynthesis pathway determines ferroptosis sensitivity in gastric cancer. Proc Natl Acad Sci U S A. 2020;117(51):32433–42. 10.1073/pnas.2006828117. 33288688 10.1073/pnas.2006828117 PMC7768719

[36] Schwab A, Rao Z, Zhang J, Gollowitzer A, Siebenkäs K, Bindel N, et al. Zeb1 mediates EMT/plasticity-associated ferroptosis sensitivity in cancer cells by regulating lipogenic enzyme expression and phospholipid composition. Nat Cell Biol. 2024;26(9):1470–81. 10.1038/s41556-024-01464-1. 39009641 10.1038/s41556-024-01464-1 PMC11392809

[37] Li M, Li Y, Yao X, Liu Y, Cai K, Yang H, et al. Optically controllable nanoregulators enable tumor-specific pro-ferroptosis lipometabolic reprogramming for in-situ adjuvant-free vaccination. Biomaterials. 2025;317:123096. 10.1016/j.biomaterials.2025.123096. 39805186 10.1016/j.biomaterials.2025.123096

[38] Xiao S, Cui J, Yang J, Hou H, Yao J, Ma X, et al. Systematic health risks assessment of chiral fungicide famoxadone: Stereoselectivities in ferroptosis-mediated cytotoxicity and metabolic behavior. J Hazard Mater. 2024;477:135199. 10.1016/j.jhazmat.2024.135199. 39053069 10.1016/j.jhazmat.2024.135199

[39] Li S, Liu H, Hu H, Ha E, Prasad P, Jenkins BC, et al. Human genetics identify convergent signals in mitochondrial LACTB-mediated lipid metabolism in cardiovascular-kidney-metabolic syndrome. Cell Metab. 2025;37(1):154-168.e7. 10.1016/j.cmet.2024.10.007. 39561766 10.1016/j.cmet.2024.10.007 PMC11972450

[40] Kamphorst JJ, Cross JR, Fan J, de Stanchina E, Mathew R, White EP, et al. Hypoxic and Ras-transformed cells support growth by scavenging unsaturated fatty acids from lysophospholipids. Proc Natl Acad Sci U S A. 2013;110(22):8882–7. 10.1073/pnas.1307237110. 23671091 10.1073/pnas.1307237110 PMC3670379

[41] Cai W, Liu L, Shi X, Liu Y, Wang J, Fang X, et al. Alox15/15-HpETE Aggravates Myocardial Ischemia-Reperfusion Injury by Promoting Cardiomyocyte Ferroptosis. Circulation. 2023;147(19):1444–60. 10.1161/circulationaha.122.060257. 36987924 10.1161/CIRCULATIONAHA.122.060257

[42] Ma XH, Liu JH, Liu CY, Sun WY, Duan WJ, Wang G, et al. ALOX15-launched PUFA-phospholipids peroxidation increases the susceptibility of ferroptosis in ischemia-induced myocardial damage. Signal Transduct Target Ther. 2022;7(1):288. 10.1038/s41392-022-01090-z. 35970840 10.1038/s41392-022-01090-z PMC9378747

[43] Lin XM, Pan MH, Sun J, Wang M, Huang ZH, Wang G, et al. Membrane phospholipid peroxidation promotes loss of dopaminergic neurons in psychological stress-induced Parkinson’s disease susceptibility. Aging Cell. 2023;22(10):e13970. 10.1111/acel.13970. 37622525 10.1111/acel.13970 PMC10577563

[44] Peng Z, Chang Y, Fan J, Ji W, Su C. Phospholipase A2 superfamily in cancer. Cancer Lett. 2021;497:165–77. 10.1016/j.canlet.2020.10.021. 33080311 10.1016/j.canlet.2020.10.021

[45] Burke JE, Dennis EA. Phospholipase A2 structure/function, mechanism, and signaling. J Lipid Res. 2009;50 Suppl(Suppl):S237–42. 10.1194/jlr.R800033-JLR200. 10.1194/jlr.R800033-JLR200 PMC2674709 19011112

[46] Murakami M, Sato H, Taketomi Y. Updating Phospholipase A(2) Biology. Biomolecules. 2020;10(10):1457. 10.3390/biom10101457. 33086624 10.3390/biom10101457 PMC7603386

[47] Sun WY, Tyurin VA, Mikulska-Ruminska K, Shrivastava IH, Anthonymuthu TS, Zhai YJ, et al. Phospholipase iPLA(2)β averts ferroptosis by eliminating a redox lipid death signal. Nat Chem Biol. 2021;17(4):465–76. 10.1038/s41589-020-00734-x. 33542532 10.1038/s41589-020-00734-x PMC8152680

[48] Chen D, Chu B, Yang X, Liu Z, Jin Y, Kon N, et al. iPLA2β-mediated lipid detoxification controls p53-driven ferroptosis independent of GPX4. Nat Commun. 2021;12(1):3644. 10.1038/s41467-021-23902-6. 34131139 10.1038/s41467-021-23902-6 PMC8206155

[49] Tyurin VA, Balasubramanian K, Winnica D, Tyurina YY, Vikulina AS, He RR, et al. Oxidatively modified phosphatidylserines on the surface of apoptotic cells are essential phagocytic “eat-me” signals: cleavage and inhibition of phagocytosis by Lp-PLA2. Cell Death Differ. 2014;21(5):825–35. 10.1038/cdd.2014.1. 24464221 10.1038/cdd.2014.1 PMC3978307

[50] Oh M, Jang SY, Lee JY, Kim JW, Jung Y, Kim J, et al. The lipoprotein-associated phospholipase A2 inhibitor Darapladib sensitises cancer cells to ferroptosis by remodelling lipid metabolism. Nat Commun. 2023;14(1):5728. 10.1038/s41467-023-41462-9. 37714840 10.1038/s41467-023-41462-9 PMC10504358

[51] Wang B, Wu L, Chen J, Dong L, Chen C, Wen Z, et al. Metabolism pathways of arachidonic acids: mechanisms and potential therapeutic targets. Signal Transduct Target Ther. 2021;6(1):94. 10.1038/s41392-020-00443-w. 33637672 10.1038/s41392-020-00443-w PMC7910446

[52] Beharier O, Tyurin VA, Goff JP, Guerrero-Santoro J, Kajiwara K, Chu T, et al. PLA2G6 guards placental trophoblasts against ferroptotic injury. Proc Natl Acad Sci U S A. 2020;117(44):27319–28. 10.1073/pnas.2009201117. 33087576 10.1073/pnas.2009201117 PMC7959495

[53] Dandavate V, Bolshette N, Van Drunen R, Manella G, Bueno-Levy H, Zerbib M, et al. Hepatic BMAL1 and HIF1α regulate a time-dependent hypoxic response and prevent hepatopulmonary-like syndrome. Cell Metab. 2024;36(9):2038-2053.e5. 10.1016/j.cmet.2024.07.003. 39106859 10.1016/j.cmet.2024.07.003

[54] Ritchie ME, Phipson B, Wu D, Hu Y, Law CW, Shi W, et al. limma powers differential expression analyses for RNA-sequencing and microarray studies. Nucleic Acids Res. 2015;43(7):e47. 10.1093/nar/gkv007. 25605792 10.1093/nar/gkv007 PMC4402510

[55] Sheng W, Zong Y, Mohammad A, Ajit D, Cui J, Han D, et al. Pro-inflammatory cytokines and lipopolysaccharide induce changes in cell morphology, and upregulation of ERK1/2, iNOS and sPLA₂-IIA expression in astrocytes and microglia. J Neuroinflammation. 2011;8:121. 10.1186/1742-2094-8-121. 21943492 10.1186/1742-2094-8-121 PMC3206447

