# Supplementary material for: Silencing arachidonate 15-lipoxygenase alleviates hypoxia-induced cognitive impairment through the mediation of phospholipase A2 group IVC–LPC18:0 to suppress ferroptosis
Source: Mol Biomed. 2026 Aug 26;7:152. doi: 10.1186/s43556-026-00546-w (PMC13518694; doi:10.1186/s43556-026-00546-w)
Supplement: Supplementary file 1 [file 43556_2026_546_MOESM1_ESM.docx]

**Supplementary information**

**Silencing arachidonate 15-lipoxygenase alleviates hypoxia-induced cognitive impairment through the mediation of phospholipase A2 group IVC–LPC18:0 to suppress ferroptosis**

# Zhen Li,^1,2,†^ Jie Gao,^1†^ Jun Fu,^1,3^ Xi Zhang,^1,3^ Yiyang Zhou,^1,3^ Chen Li,^1^ Jutaek Nam,^2*^ Hongchang Gao,^1,3*^

# ^1^School of Pharmaceutical Sciences, Oujiang Laboratory (Zhejiang Lab for Regenerative Medicine, Vision and Brain Health), Institute of Metabonomics & Medical NMR, Wenzhou Medical University, Wenzhou 325035, China

# ^2^College of Pharmacy, Chonnam National University, Gwangju 61186, Republic of Korea

# ^3^Innovation Academy of Testing Technology, Research and Experiment Center, Wenzhou Medical University, Wenzhou 325035, China

# ^*^Correspond: Jutaek Nam (namj@jnu.ac.kr); Hongchang Gao (gaohc27@wmu.edu.cn)

# ^†^These authors contributed equally to this work.

**Fig. S1.** Supplementation of ferrostatin-1 alleviates hypoxia (HYP)-mediated cognitive impairment. Mice in the treatment group received ferrostatin-1 at 1 mg/kg by intraperitoneal injection for 21 days.

(a) The escape latency of mice during the positioning navigation test.
(b) The swimming trajectories.
(c) The swimming distance percentages.
(d) The time in the goal area.
(e) The number of crossings over the original platform location.

(f) The total distance of exploration of the mice in the spatial exploration test. Data are shown as the mean ± SD (n = 6 per group). The differences among groups were analyzed using one-way ANOVA followed by Tukey’s post hoc test. Statistical significance was defined as **p* < 0.05, ***p* < 0.01, and ****p* < 0.001; ns, not significant.

**Fig. S2.** The mRNA levels of rat hippocampal central node genes in

(a) the 1-day group;
(b) the 3-day group;

(c) the 7-day group compared with the control group. Data are shown as the mean ± SD (n = 3 per group). Student’s t-test was used to assess statistical significance. Statistical significance was defined as **p <* 0.05; ***p <* 0.01; ns, not significant.

**Fig. S3.** SH-SY5Y cells were subjected to hypoxia for 8 or 12 h.

(a) Cell viability was measured by CCK-8 assay (*n* = 6).

(b) The mRNA levels of *GPX4*, *ACSL4*, and *SLC7A11* in cells **across varying durations of HYP** (n = 3)**.**

(c) Representative Western blot images and quantitative analysis of ALOX15 and GPX4 protein expression in cells after 8 h of HYP. Data are presented as the mean ± SD from at least three independent experiments. The differences among the groups were analyzed using one-way ANOVA with Tukey’s post hoc test. Significance was determined at a *p*-level of **p <* 0.05; ***p <* 0.01; ****p* < 0.001; ns, not significant.

**Fig. S4.** Pharmacological validation of ferroptosis involvement in hypoxia-induced SH-SY5Y cell injury. SH-SY5Y cells were exposed to hypoxia and treated with ferroptosis-related inhibitors, including Ferrostatin-1 (Fer-1), Deferasirox (DFX), and Liproxstatin-1.

(a) Cell viability was measured by CCK-8 assay (*n* = 6).

(b, c) Intracellular MDA and GSH contents in SH-SY5Y cells subjected to the indicated treatments. MDA content was normalized to total protein and expressed as nmol/mg protein, whereas GSH content was normalized to the Control group and expressed as relative GSH content (*n* = 6).

(d) Lipid-mediated ROS production was detected by flow cytometry after staining SH-SY5Y cells with BODIPY.

(e) Quantitative analysis of lipid peroxidation levels (*n* = 3). Data are presented as mean ± SD. **p* < 0.05, ***p* < 0.01, ****p* < 0.001, *****p* < 0.0001; ns, not significant.

**Fig. S5.** Glutathione peroxidase 4 overexpression rescues hypoxia- or arachidonate 15-lipoxygenase overexpression-induced ferroptosis-related injury in SH-SY5Y cells.
(a) Representative Western blot images.

(b) Quantitative analysis of GPX4 protein expression (*n* = 3).

(c) Cell viability was measured by CCK-8 assay (*n* = 6).

(d, e) Intracellular MDA and GSH levels in SH-SY5Y cells exposed to HYP, HYP combined with GPX4 overexpression (HYP + OE GPX4), ALOX15 overexpression (OE ALOX15), or combined ALOX15 and GPX4 overexpression (OE ALOX15 + OE GPX4). MDA content was normalized to total protein and expressed as nmol/mg protein, whereas GSH content was expressed relative to the corresponding HYP or OE ALOX15 reference group (n = 6).

(f) Lipid-mediated ROS production was detected by flow cytometry after staining SH-SY5Y cells with BODIPY.

(g) Quantitative analysis of lipid peroxidation levels (*n* = 3). Data are presented as mean ± SD. **p* < 0.05, ***p* < 0.01, ****p* < 0.001, *****p* < 0.0001; ns, not significant.

**Fig. S6.** BV2 cells were exposed to hypoxia for 12 h with or without arachidonate 15-lipoxygenase knockdown. Conditioned medium (CM) from BV2 cells was then collected and used to treat SH-SY5Y cells under hypoxia.

(a) Illustration of the generation of CM.
(b) ALOX15 protein levels in SH-SY5Y cells (*n* = 3).
(c) *Il6*, *Il1β*, and *Il10* mRNA expression levels in BV2 cells (*n* = 3).

(d) SH-SY5Y cells treated with LPS (100 ng/mL) for 24 h and then treated with IL-10 (10 ng/mL) for an additional 24 h; ALOX15 protein levels were then measured (*n* = 3). Data are shown as the mean ± SD of at least three independent experiments. The differences among groups were analyzed using one-way ANOVA with Tukey’s post hoc test or Student’s t-test. Statistical significance was defined as **p* < 0.05; ***p* < 0.01; ns, not significant.

**Fig. S7.** Arachidonate 15-lipoxygenase-regulated hypoxia (HYP) injury is associated with phospholipase A2 group IVC suppression.

(a) Relative PLA2G4C mRNA expression in HYP-treated SH-SY5Y cells with or without ALOX15 knockdown (*n* = 3).
(b) PLA2G4C protein expression in HYP-treated SH-SY5Y cells after *ALOX15* knockdown or *ALOX15* overexpression (*n* = 3).
(c) Representative Western blot images and quantitative analysis of ALOX15 protein expression in SH-SY5Y cells transfected with either *PLA2G4C* overexpression plasmid or control plasmid (*n* = 3).

(d) SH-SY5Y cells were transfected with shRNA targeting *PLA2G4C* and *ALOX15*. Representative Western blot images and quantitative analysis of ALOX15 protein expression in SH-SY5Y cells (n = 3).

(e) PLA2G4C and GPX4 protein levels in SH-SY5Y cells transfected with *PLA2G4C* overexpression plasmid or control plasmid under HYP (*n* = 3). Data are shown as the mean ± SD of at least three independent experiments. The differences among groups were analyzed using one-way ANOVA with Tukey’s post hoc test or Student’s t-test. tatistical significance was defined as **p* < 0.05; ***p* < 0.01; ns, not significant.

**Fig. S8**. Phospholipase A2 group IVC overexpression partially rescues arachidonate 15-lipoxygenase overexpression-induced ferroptosis-related alterations under hypoxia.
(a) Representative Western blot images of GPX4, ALOX15, PLA2G4C, and β-actin in HYP-treated cells after *ALOX15* overexpression, *PLA2G4C* overexpression, or combined *ALOX15/PLA2G4C* overexpression.

(b–d) Quantitative analysis of PLA2G4C, ALOX15, and GPX4 protein expression (*n* = 3).

(e, f) Quantification of MDA and GSH levels in each group (*n* = 3). The differences among groups were analyzed using one-way ANOVA with Tukey’s post hoc test. Significance was determined at a *p*-level of **p* < 0.05; ***p* < 0.01; ns, not significant.

**Fig. S9.** MAFK participates in arachidonate 15-lipoxygenase -mediated regulation of phospholipase A2 group IVC transcription.

(a) Candidate transcription factors of PLA2G4C were screened by RNA-seq, GTRD, and JASPAR analyses.

(b) RT-qPCR validation of CREM and MAFK expression after ALOX15 inhibition.

(c) Knockdown efficiency of si-*CREM* and si-*MAFK*.

(d) PLA2G4C mRNA expression after *CREM* or *MAFK* knockdown.

(e, f) Time-course changes in MAFK and CREM expression after ALOX15 inhibition.

(g) Dual-luciferase reporter assay results for MAFK and the full-length PLA2G4C promoter.

(h) PLA2G4C promoter truncation analysis. (i) PLA2G4C mRNA expression after *ALOX15* and/or *MAFK* overexpression. (j) Effect of *ALOX15* overexpression on MAFK-induced PLA2G4C promoter activity. Data are presented as mean ± SD. ns, not significant; **p* < 0.05, ***p* < 0.01, ****p* < 0.001, *****p* < 0.0001.

**Fig. S10.** LPC18:0 concentrations in serum and hippocampal tissues and preliminary *in vitro* blood–brain barrier permeability assessment. (a) Serum LPC18:0 concentration-time profile over 24 h. (b) LPC18:0 content in hippocampal tissues at the indicated time points. (c) Schematic illustration of the bEnd.3 cell-based Transwell model. (d) LPC18:0 concentrations in the lower chamber of the Transwell system at 0, 1, 2, and 4 h. Data are presented as mean ± SD.

**Fig. S11.** Serum lipidomic changes after LPC18:0 supplementation in hypoxia-exposed mice. Heatmap analysis showing the relative abundance of (a) LPC, (b) PC, and (c) PE species among the CON, HYP, and HYP + LPC18:0 groups. The color scale represents normalized relative abundance, with red indicating higher levels and blue indicating lower levels.

**Fig. S12.** Experimental design and bioinformatic workflow.

(a) Timeline for the HYP-induced cognitive impairment mouse model, (b) timeline for hippocampal AAV-mediated Alox15 knockdown, (c) timeline for LPC18:0 administration in HYP-exposed mice, (d) timeline of rat hippocampal samples used for HYP-related gene validation at 1, 3, and 7 days. (e) Bioinformatic workflow for screening ferroptosis-related hub genes using GEO and FerrDb datasets.

**Table S1**. Primers used for quantitative real-time polymerase chain reaction.

| Primer | Sense/antisense | Sequence |
| --- | --- | --- |
| Rat *Hmox1* | Forward | AGGGTCAGGTGTCCAGGGAAG |
|  | Reverse | TCTGCTTGTTTCGCTCTATCTCCTC |
| Rat *Alox15* | Forward | CTTCCTGCCCGCCTGGTATTC |
|  | Reverse | CCGCTTCAAACAGAGTGCCTTTC |
| Rat *Il1β* | Forward | TCTCACAGCAGCATCTCGACAAG |
|  | Reverse | CCACGGGCAAGACATAGGTAGC |
| Rat *Cdkn1a* | Forward | CCCGTGGACAGTGAGCAGTTG |
|  | Reverse | GGCGTCTCAGTGGCGAAGTC |
| Rat *Adipoq* | Forward | GACGGCAGCACTGGCAAATTC |
|  | Reverse | CCTGATACTGGTCGTAGGTGAAGAG |
| Rat *Cdh1* | Forward | GCCTACACCATCCTCAGCCAAG |
|  | Reverse | AGGGTAACTCTCTCGGTCCAGTC |
| Rat *Lcn2* | Forward | CTGACTACGACCAGTTTGCCATG |
|  | Reverse | GACGAATCGCTCCTTCAGTTCATC |
| Rat *Nos2* | Forward | TTGGAGCGAGTTGTGGATTGTTC |
|  | Reverse | AGTGATGTCCAGGAAGTAGGTGAG |
| Rat *Atf2* | Forward | CTCACCAGGATAGCCCGTTACC |
|  | Reverse | AACCTGTAATGATGCTGGACGAAC |
| Rat *Hspb1* | Forward | ACTCAGCAGCGGTGTCTCAG |
|  | Reverse | TCTCCACCACGCCTTCCTTG |
| Rat *Tlr4* | Forward | GAATGAGGACTGGGTGAGAAACG |
|  | Reverse | TGGATGATGTTGGCAGCAATGG |
| Human *PLA2G4C* | Forward | GGTTCACCTCATCCTCTCCTTCG |
|  | Reverse | GGCGGCGGCAGTAGTCAG |
| Human *GPX4* | Forward | GCTGTGGAAGTGGATGAA |
|  | Reverse | GATGAGGAACTGTGGAGAG |
| Human *ACSL4* | Forward | TGGTTCTACTGGCCGACCTA |
|  | Reverse | TCGGTCCCAGTCCAGGTATT |
| Human *SLC7A11* | Forward | CCTGTTGTGTCCACCATC |
|  | Reverse | AAGATTCCTGCTCCAATGAT |
| Human *CREM* | Forward | ACAGTACGCAGCACAATCAGC |
|  | Reverse | GGATCTGGTAAGTTGGCATGTCAC |
| Human *MAFK* | Forward | TGACGACTAATCCCAAACCGAATAAG |
|  | Reverse | GACATGGACACCAGCTCATCATC |
| Mouse *Il6* | Forward | TCCATCCAGTTGCCTTCT |
|  | Reverse | TAAGCCTCCGACTTGTGA |
| Mouse *Il1β* | Forward | CTTCAGGCAGGCAGTATC |
|  | Reverse | CAGCAGGTTATCATCATCATC |
| Mouse *Il10* | Forward | GGTTGCCAAGCCTTATCG |
|  | Reverse | TCTTCACCTGCTCCACTG |

**Table S2**. Multiple reaction monitoring transition parameters for phospholipid/lysophospholipid lipidomic analysis.

| Precursor m/z | Product m/z | Name | Formula | Retention time [min] | Dwell time [ms] | Q1 Pre bias [V] | Collision energy [V] | Q3 Pre bias [V] |
| --- | --- | --- | --- | --- | --- | --- | --- | --- |
| 468.3 | 184.1 | lysoPC 14:0 (sn-1) | C22H46NO7P | 7.03 | 5 | 20 | 20 | 25 |
| 482.3 | 184.1 | lysoPC 15:0 (sn-1) | C23H48NO7P | 8.65 | 5 | 20 | 20 | 25 |
| 480.3 | 184.1 | lysoPC 15:1 (sn-1) | C23H48NO7P | 7.23 | 5 | 20 | 20 | 25 |
| 496.3 | 184.1 | lysoPC 16:0 (sn-1) | C24H50NO7P | 10.40 | 5 | 20 | 20 | 25 |
| 482.3 | 184.1 | lysoPC 16:0e | C24H52NO6P | 12.20 | 5 | 20 | 20 | 25 |
| 480.3 | 184.1 | lysoPC 16:0p | C24H50NO6P | 11.86 | 5 | 20 | 20 | 25 |
| 494.3 | 184.1 | lysoPC 16:1 (sn-1) | C24H48NO7P | 8.00 | 5 | 20 | 20 | 25 |
| 510.4 | 184.1 | lysoPC 17:0 (sn-1) | C25H52NO7P | 12.10 | 5 | 20 | 20 | 25 |
| 508.3 | 184.1 | lysoPC 17:1 (sn-1) | C25H50NO7P | 9.62 | 5 | 20 | 20 | 25 |
| 524.4 | 184.1 | lysoPC 18:0 (sn-1) | C26H54NO7P | 13.72 | 5 | 20 | 20 | 25 |
| 508.3 | 184.1 | lysoPC 18:0p | C26H54NO6P | 15.07 | 5 | 20 | 20 | 25 |
| 522.4 | 184.1 | lysoPC 18:1 (sn-1) | C26H52NO7P | 11.27 | 5 | 20 | 20 | 25 |
| 520.3 | 184.1 | lysoPC 18:2 (sn-1) | C26H50NO7P | 9.27 | 5 | 20 | 20 | 25 |
| 518.3 | 184.1 | lysoPC 18:3 (sn-1) | C26H48NO7P | 7.66 | 5 | 20 | 20 | 25 |
| 538.4 | 184.1 | lysoPC 19:0 (sn-1) | C27H56NO7P | 15.27 | 5 | 20 | 20 | 25 |
| 552.4 | 184.1 | lysoPC 20:0 (sn-1) | C28H58NO7P | 16.67 | 5 | 20 | 20 | 25 |
| 550.4 | 184.1 | lysoPC 20:1 (sn-1) | C28H56NO7P | 14.36 | 5 | 20 | 20 | 25 |
| 548.4 | 184.1 | lysoPC 20:2 (sn-1) | C28H54NO7P | 12.38 | 5 | 20 | 20 | 25 |
| 546.4 | 184.1 | lysoPC 20:3 (sn-1) | C28H52NO7P | 10.57 | 5 | 20 | 20 | 25 |
| 544.3 | 184.1 | lysoPC 20:4 (sn-1) | C28H50NO7P | 9.29 | 5 | 20 | 20 | 25 |
| 542.3 | 184.1 | lysoPC 20:5 (sn-1) | C28H48NO7P | 7.70 | 5 | 20 | 20 | 25 |
| 580.4 | 184.1 | lysoPC 22:0 (sn-1) | C30H62NO7P | 19.17 | 5 | 20 | 20 | 25 |
| 572.4 | 184.1 | lysoPC 22:4 (sn-1) | C30H54NO7P | 11.92 | 5 | 20 | 20 | 25 |
| 568.3 | 184.1 | lysoPC 22:6 (sn-1) | C30H50NO7P | 9.33 | 5 | 20 | 20 | 25 |
| 622.4 | 184.1 | PC 24:0; PC 12:0/12:0 (IS) | C32H64NO8P | 17.70 | 5 | 24 | 26 | 26 |
| 706.5 | 184.1 | PC 30:0; PC 14:0/16:0 | C38H76NO8P | 23.39 | 5 | 24 | 26 | 26 |
| 704.5 | 184.1 | PC 30:1; PC 14:0/16:1 | C38H74NO8P | 22.16 | 5 | 24 | 26 | 26 |
| 720.5 | 184.1 | PC 31:0; PC 16:0e/16:0 | C39H80NO7P | 26.02 | 5 | 24 | 26 | 26 |
| 718.5 | 184.1 | PC 31:1p; PC 16:0p/16:0 | C39H76NO7P | 25.72 | 5 | 24 | 26 | 26 |
| 734.6 | 184.1 | PC 32:0; PC 16:0/16:0 | C40H80NO8P | 24.97 | 5 | 24 | 26 | 26 |
| 732.5 | 184.1 | PC 32:1; PC 14:0/18:1 or 16:0/16:1 | C40H78NO8P | 23.74 | 5 | 24 | 26 | 26 |
| 730.5 | 184.1 | PC 32:2; PC 14:0/18:2 or 16:1/16:1 | C40H76NO8P | 22.69 | 5 | 24 | 26 | 26 |
| 748.6 | 184.1 | PC 33:0e; PC 18:0e/16:0 | C41H84NO7P | 27.88 | 5 | 24 | 26 | 26 |
| 746.6 | 184.1 | PC 33:1e; PC 18:0e/16:1 or 18:1e/16:0 | C41H82NO7P | 26.32 | 5 | 24 | 26 | 26 |
| 744.6 | 184.1 | PC 33:2e; PC 16:1e/18:1 | C41H80NO7P | 25.23 | 5 | 24 | 26 | 26 |
| 762.6 | 184.1 | PC 34:0; PC 16:0/18:0 | C42H84NO8P | 26.67 | 5 | 24 | 26 | 26 |
| 760.6 | 184.1 | PC 34:1; PC 16:0/18:1 | C42H82NO8P | 25.27 | 5 | 24 | 26 | 26 |
| 758.6 | 184.1 | PC 34:2; PC 16:0/18:2 or 16:1/18:1 | C42H80NO8P | 24.22 | 5 | 24 | 26 | 26 |
| 756.6 | 184.1 | PC 34:3; PC 16:0/18:3 or 16:1/18:2 | C42H78NO8P | 23.41 | 5 | 24 | 26 | 26 |
| 754.5 | 184.1 | PC 34:4; PC 14:0/20:4 or 16:1/18:3 | C42H76NO8P | 22.62 | 5 | 24 | 26 | 26 |
| 752.5 | 184.1 | PC 34:5; PC 14:0/20:5 | C42H74NO8P | 21.74 | 5 | 24 | 26 | 26 |
| 774.6 | 184.1 | PC 35:1; PC 16:0/19:1 or 17:0/18:1 or 17:1/18:0 | C43H84NO8P | 26.06 | 5 | 24 | 26 | 26 |
| 772.6 | 184.1 | PC 35:2e; PC 18:0p/18:1 or 18:1e/18:1 | C43H84NO7P | 26.67 | 5 | 24 | 26 | 26 |
| 770.6 | 184.1 | PC 35:3e; PC 18:2e/18:1 | C43H82NO7P | 25.80 | 5 | 24 | 26 | 26 |
| 768.6 | 184.1 | PC 35:4; PC 16:1e/20:3 | C43H78NO8P | 25.11 | 5 | 24 | 26 | 26 |
| 766.5 | 184.1 | PC 35:5p; PC 16:0p/20:4 | C43H76NO7P | 24.83 | 5 | 24 | 26 | 26 |
| 790.6 | 184.1 | PC 36:0; PC 18:0/18:0 | C44H88NO8P | 28.66 | 5 | 24 | 26 | 26 |
| 788.6 | 184.1 | PC 36:1; PC 18:0/18:1 | C44H86NO8P | 27.07 | 5 | 24 | 26 | 26 |
| 786.6 | 184.1 | PC 36:2; PC 18:0/18:2 or 18:1/18:1 | C44H84NO8P | 25.82 | 5 | 24 | 26 | 26 |
| 784.6 | 184.1 | PC 36:3; PC 16:0/20:3 or 18:0/18:3 or 18:1/18:2 | C44H82NO8P | 24.66 | 5 | 24 | 26 | 26 |
| 782.6 | 184.1 | PC 36:4; PC 18:1/18:3 or 18:2/18:2 | C44H80NO8P | 23.55 | 5 | 24 | 26 | 26 |
| 780.6 | 184.1 | PC 36:5; PC 18:2/18:3 | C44H78NO8P | 22.93 | 5 | 24 | 26 | 26 |
| 778.5 | 184.1 | PC 36:6; PC 16:1/20:5 | C44H76NO8P | 22.14 | 5 | 24 | 26 | 26 |
| 802.6 | 184.1 | PC 37:1; PC 18:0/19:1 or 19:0/18:1 | C45H88NO8P | 27.96 | 5 | 24 | 26 | 26 |
| 800.6 | 184.1 | PC 37:2; PC 18:1/19:1 or 18:2/19:0 | C45H86NO8P | 26.70 | 5 | 24 | 26 | 26 |
| 798.6 | 184.1 | PC 37:3; PC 17:0/20:3 or 19:1/18:2 | C45H84NO8P | 25.59 | 5 | 24 | 26 | 26 |
| 796.6 | 184.1 | PC 37:4; PC 17:0/20:4 | C45H82NO8P | 24.91 | 5 | 24 | 26 | 26 |
| 796.6 | 184.1 | PC 37:4e; PC 18:0e/20:4 | C45H84NO7P | 26.85 | 5 | 24 | 26 | 26 |
| 794.6 | 184.1 | PC 37:5; PC 17:0/20:5 or 17:1/20:4 | C45H80NO8P | 24.10 | 5 | 24 | 26 | 26 |
| 792.6 | 184.1 | PC 37:6; PC 15:0/22:6 | C45H78NO8P | 23.29 | 5 | 24 | 26 | 26 |
| 816.6 | 184.1 | PC 38:1; PC 18:1/20:0 | C46H90NO8P | 29.01 | 5 | 24 | 26 | 26 |
| 814.6 | 184.1 | PC 38:2; PC 16:0/22:2 | C46H88NO8P | 27.22 | 5 | 24 | 26 | 26 |
| 812.6 | 184.1 | PC 38:3; PC 18:0/20:3 or 18:1/20:2 | C46H86NO8P | 26.44 | 5 | 24 | 26 | 26 |
| 810.6 | 184.1 | PC 38:4; PC 18:0/20:4 | C46H84NO8P | 25.73 | 5 | 24 | 26 | 26 |
| 808.6 | 184.1 | PC 38:5; PC 18:0/20:5 | C46H82NO8P | 24.86 | 5 | 24 | 26 | 26 |
| 806.6 | 184.1 | PC 38:6; PC 16:0/22:6 or 18:2/20:4 | C46H80NO8P | 24.02 | 5 | 24 | 26 | 26 |
| 804.6 | 184.1 | PC 38:7; PC 16:1/22:6 or 18:2/20:5 | C46H78NO8P | 22.62 | 5 | 24 | 26 | 26 |
| 826.6 | 184.1 | PC 39:3; PC 19:0/20:3 | C47H88NO8P | 27.33 | 5 | 24 | 26 | 26 |
| 820.6 | 184.1 | PC 39:6; PC 17:0/22:6 | C47H84NO8P | 24.76 | 5 | 24 | 26 | 26 |
| 818.6 | 184.1 | PC 39:7; PC 17:1/22:6 | C47H80NO8P | 24.18 | 5 | 24 | 26 | 26 |
| 844.7 | 184.1 | PC 40:1; PC 18:1/22:0 | C48H94NO8P | 31.51 | 5 | 24 | 26 | 26 |
| 838.6 | 184.1 | PC 40:4; PC 18:0/22:4 or 20:1/20:3 | C48H88NO8P | 27.57 | 5 | 24 | 26 | 26 |
| 836.6 | 184.1 | PC 40:5; PC 18:0/22:5 | C48H86NO8P | 26.54 | 5 | 24 | 26 | 26 |
| 834.6 | 184.1 | PC 40:6; PC 18:0/22:6 | C48H84NO8P | 25.59 | 5 | 24 | 26 | 26 |
| 832.6 | 184.1 | PC 40:7; PC 18:1/22:6 | C48H82NO8P | 24.28 | 5 | 24 | 26 | 26 |
| 832.6 | 184.1 | PC 40:7; PC 20:3/20:4 | C48H82NO8P | 23.91 | 5 | 24 | 26 | 26 |
| 830.6 | 184.1 | PC 40:8; PC 20:4/20:4 | C48H80NO8P | 23.27 | 5 | 24 | 26 | 26 |
| 848.6 | 184.1 | PC 41:6; PC 19:0/22:6 | C49H86NO8P | 26.42 | 5 | 24 | 26 | 26 |
| 426.3 | 285.3 | lysoPE 14:0 (sn-2) | C19H40NO7P | 6.40 | 5 | 17 | 15 | 25 |
| 454.3 | 313.3 | lysoPE 16:0 (sn-1) | C21H44NO7P | 10.52 | 5 | 17 | 15 | 25 |
| 468.3 | 327.3 | lysoPE 17:0 (sn-1) | C22H46NO7P | 12.22 | 5 | 17 | 15 | 25 |
| 482.3 | 341.3 | lysoPE 18:0 (sn-1) | C23H48NO7P | 13.83 | 5 | 17 | 15 | 25 |
| 468.3 | 327.3 | lysoPE 18:0e | C23H50NO6P | 15.47 | 5 | 17 | 15 | 25 |
| 480.3 | 339.3 | lysoPE 18:1 (sn-1) | C23H46NO7P | 11.35 | 5 | 17 | 15 | 25 |
| 478.3 | 337.3 | lysoPE 18:2 (sn-1) | C23H44NO7P | 9.37 | 5 | 17 | 15 | 25 |
| 510.4 | 369.4 | lysoPE 20:0 (sn-1) | C25H52NO7P | 16.74 | 5 | 17 | 15 | 25 |
| 508.3 | 367.3 | lysoPE 20:1 (sn-1) | C25H50NO7P | 14.42 | 5 | 17 | 15 | 25 |
| 504.3 | 363.3 | lysoPE 20:3 (sn-1) | C25H46NO7P | 10.70 | 5 | 17 | 15 | 25 |
| 502.3 | 361.3 | lysoPE 20:4 (sn-1) | C25H44NO7P | 9.35 | 5 | 17 | 15 | 25 |
| 500.3 | 359.3 | lysoPE 20:5 (sn-2) | C25H42NO7P | 7.77 | 5 | 17 | 15 | 25 |
| 526.3 | 385.3 | lysoPE 22:6 (sn-2) | C27H44NO7P | 9.51 | 5 | 17 | 15 | 25 |
| 692.5 | 551.5 | PE 32:0; PE 16:0/16:0 | C37H74NO8P | 24.94 | 5 | 20 | 20 | 30 |
| 690.5 | 549.5 | PE 32:1; PE 14:0/18:1 or 16:0/16:1 | C37H72NO8P | 23.73 | 5 | 20 | 20 | 30 |
| 688.5 | 547.5 | PE 32:2; PE 14:0/18:2 | C37H70NO8P | 22.60 | 5 | 20 | 20 | 30 |
| 704.6 | 563.6 | PE 33:1e ; PE 16:0e/18:1 | C38H76NO7P | 26.32 | 5 | 20 | 20 | 30 |
| 702.5 | 561.5 | PE 33:2; PE 15:0/18:2 | C38H72NO8P | 23.27 | 5 | 20 | 20 | 30 |
| 720.6 | 579.6 | PE 34:0; PE 16:0/18:0 | C39H78NO8P | 26.56 | 5 | 20 | 20 | 30 |
| 718.5 | 577.5 | PE 34:1; PE 16:0/18:1 | C39H76NO8P | 25.21 | 5 | 20 | 20 | 30 |
| 716.5 | 575.5 | PE 34:2; PE 16:0/18:2 or 16:1/18:1 | C39H74NO8P | 24.26 | 5 | 20 | 20 | 30 |
| 714.5 | 573.5 | PE 34:3; PE 16:0/18:3 | C39H72NO8P | 23.00 | 5 | 20 | 20 | 30 |
| 732.6 | 591.6 | PE 35:1; PE 17:0/18:1 | C40H78NO8P | 26.05 | 5 | 20 | 20 | 30 |
| 730.5 | 589.5 | PE 35:2; PE 17:0/18:2 | C40H76NO8P | 25.04 | 5 | 20 | 20 | 30 |
| 728.5 | 587.5 | PE 35:3; PE 17:1/18:2 | C40H74NO8P | 24.00 | 5 | 20 | 20 | 30 |
| 726.5 | 585.5 | PE 35:4p; PE 16:0p/20:3 or 16:0e/20:4 | C40H72NO7P | 25.14 | 5 | 20 | 20 | 30 |
| 724.5 | 583.5 | PE 35:5p; PE 16:0p/20:4 | C40H70NO7P | 24.80 | 5 | 20 | 20 | 30 |
| 722.5 | 581.5 | PE 35:6p; PE 16:0p/20:5 | C40H68NO7P | 23.70 | 5 | 20 | 20 | 30 |
| 746.6 | 605.6 | PE 36:1; PE 16:0/20:1 or 18:0/18:1 | C41H80NO8P | 27.00 | 5 | 20 | 20 | 30 |
| 744.6 | 603.6 | PE 36:2; PE 18:0/18:2 or 18:1/18:1 | C41H78NO8P | 25.79 | 5 | 20 | 20 | 30 |
| 742.5 | 601.5 | PE 36:3; PE 18:1/18:2 | C41H76NO8P | 24.70 | 5 | 20 | 20 | 30 |
| 740.5 | 599.5 | PE 36:4; PE 16:0/20:4 | C41H74NO8P | 24.15 | 5 | 20 | 20 | 30 |
| 738.5 | 597.5 | PE 36:5; PE 16:0/20:5 | C41H72NO8P | 23.33 | 5 | 20 | 20 | 30 |
| 736.5 | 595.5 | PE 36:6; PE 16:1/20:5 | C41H70NO8P | 22.00 | 5 | 20 | 20 | 30 |
| 758.6 | 617.6 | PE 37:2; PE 19:0/18:2 | C42H80NO8P | 26.60 | 5 | 20 | 20 | 30 |
| 756.6 | 615.6 | PE 37:3; PE 17:0/20:3 | C42H78NO8P | 25.50 | 5 | 20 | 20 | 30 |
| 754.5 | 613.5 | PE 37:4; PE 17:0/20:4 | C42H76NO8P | 24.94 | 5 | 20 | 20 | 30 |
| 754.5 | 613.5 | PE 37:4e ; PE 18:1e/20:3 | C42H78NO7P | 26.38 | 5 | 20 | 20 | 30 |
| 752.5 | 611.5 | PE 37:5; PE 17:1/20:4 | C42H74NO8P | 23.30 | 5 | 20 | 20 | 30 |
| 752.5 | 611.5 | PE 37:5p; PE 18:0p/20:4 | C42H74NO7P | 26.48 | 5 | 20 | 20 | 30 |
| 750.5 | 609.5 | PE 37:6p; PE 18:0p/20:5 or 18:1p/20:4 or 16:0e/22:6 | C42H72NO7P | 24.92 | 5 | 20 | 20 | 30 |
| 748.6 | 607.6 | PE 37:7p; PE 16:0p/22:6 | C42H70NO7P | 24.64 | 5 | 20 | 20 | 30 |
| 774.6 | 633.6 | PE 38:1; PE 18:0/20:1 | C43H84NO8P | 28.94 | 5 | 20 | 20 | 30 |
| 772.6 | 631.6 | PE 38:2; PE 20:0/18:2 | C43H82NO8P | 27.49 | 5 | 20 | 20 | 30 |
| 770.6 | 629.6 | PE 38:3; PE 18:0/20:3 | C43H80NO8P | 26.39 | 5 | 20 | 20 | 30 |
| 768.6 | 627.6 | PE 38:4; PE 16:0/22:4 | C43H78NO8P | 25.06 | 5 | 20 | 20 | 30 |
| 768.6 | 627.6 | PE 38:4; PE 18:0/20:4 | C43H78NO8P | 25.69 | 5 | 20 | 20 | 30 |
| 768.6 | 627.6 | PE 38:4; PE 18:1/20:3 or 18:2/20:2 | C43H78NO8P | 25.32 | 5 | 20 | 20 | 30 |
| 766.5 | 625.5 | PE 38:5; PE 18:0/20:5 | C43H76NO8P | 24.86 | 5 | 20 | 20 | 30 |
| 764.5 | 623.5 | PE 38:6; PE 16:0/22:6 or 16:1/22:5 or 20:2/18:4 | C43H74NO8P | 23.97 | 5 | 20 | 20 | 30 |
| 762.5 | 621.5 | PE 38:7; PE 16:1/22:6 or 18:2/20:5 | C43H72NO8P | 22.40 | 5 | 20 | 20 | 30 |
| 782.6 | 641.6 | PE 39:4; PE 19:0/20:4 | C44H80NO8P | 26.11 | 5 | 20 | 20 | 30 |
| 780.6 | 639.6 | PE 39:5; PE 17:0/22:5 | C44H78NO8P | 25.06 | 5 | 20 | 20 | 30 |
| 778.5 | 637.5 | PE 39:6; PE 17:0/22:6 | C44H76NO8P | 24.50 | 5 | 20 | 20 | 30 |
| 776.5 | 635.5 | PE 39:7; PE 17:1/22:6 | C44H74NO8P | 23.36 | 5 | 20 | 20 | 30 |
| 802.6 | 661.6 | PE 40:1; PE 22:0/18:1 | C45H88NO8P | 30.20 | 5 | 20 | 20 | 30 |
| 800.6 | 659.6 | PE 40:2; PE 18:1/22:1 | C45H86NO8P | 29.00 | 5 | 20 | 20 | 30 |
| 798.6 | 657.6 | PE 40:3; PE 18:1/22:2 or 22:1/18:2 | C45H84NO8P | 28.00 | 5 | 20 | 20 | 30 |
| 796.6 | 655.6 | PE 40:4; PE 18:0/22:4 or 20:0/20:4 | C45H82NO8P | 27.02 | 5 | 20 | 20 | 30 |
| 794.6 | 653.6 | PE 40:5; PE 18:0/22:5 | C45H80NO8P | 26.03 | 5 | 20 | 20 | 30 |
| 792.6 | 651.6 | PE 40:6; PE 18:1/22:5 | C45H78NO8P | 25.15 | 5 | 20 | 20 | 30 |
| 790.5 | 649.5 | PE 40:7; PE 18:1/22:6 | C45H76NO8P | 24.35 | 5 | 20 | 20 | 30 |
| 822.6 | 681.6 | PE 42:5; PE 22:1/20:4 | C47H84NO8P | 28.00 | 5 | 20 | 20 | 30 |
| 818.6 | 677.6 | PE 42:7; PE 20:1/22:6 | C47H80NO8P | 26.00 | 5 | 20 | 20 | 30 |
| 816.6 | 675.6 | PE 42:8; PE 20:2/22:6 | C47H78NO8P | 25.00 | 5 | 20 | 20 | 30 |
| 199 | 199 | FFA 12:0 (Lauric acid) | C12H24O2 | 4.53 | 5 | 25 | 10 | 25 |
| 213.2 | 213.2 | FFA 13:0 (Tridecanoic acid) | C13H26O2 | 5.50 | 5 | 17 | 10 | 20 |
| 227.1 | 227.1 | FFA 14:0 (Myristic acid) | C14H28O2 | 7.52 | 5 | 15 | 10 | 15 |
| 225.2 | 225.2 | FFA 14:1 (Myristoleic acid, n–5) | C14H26O2 | 5.44 | 5 | 15 | 10 | 15 |
| 240.8 | 240.8 | FFA 15:0 (Pentadecylic acid) | C15H30O2 | 9.10 | 5 | 16 | 10 | 16 |
| 255.05 | 255.05 | FFA 16:0 (Palmitic acid) | C16H32O2 | 11.02 | 5 | 17 | 10 | 17 |
| 253.1 | 253.1 | FFA 16:1 (Palmitoleic acid, n–7) | C16H30O2 | 10.59 | 5 | 15 | 10 | 15 |
| 268.9 | 268.9 | FFA 17:0 (Margaric acid) | C17H34O2 | 12.75 | 5 | 13 | 10 | 13 |
| 267.2 | 267.2 | FFA 17:1 (cis-10-Heptadecanoic acid, n–7) | C17H32O2 | 10.25 | 5 | 12 | 10 | 12 |
| 283.05 | 283.05 | FFA 18:0 (Stearic acid) | C18H36O2 | 14.45 | 5 | 18 | 10 | 15 |
| 280.9 | 280.9 | FFA 18:1 (Oleic acid, n–9 or cis-Vaccenic acid, n–7) | C18H34O2 | 11.86 | 5 | 12 | 10 | 12 |
| 280.9 | 280.9 | FFA 18:1 (Elaidic acid, n–9 or trans-Vaccenic acid, n–7) | C18H34O2 | 12.40 | 5 | 19 | 10 | 19 |
| 278.95 | 278.95 | FFA 18:2 (Linoleic acid, n–6) | C18H32O2 | 9.76 | 5 | 21 | 10 | 21 |
| 276.9 | 276.9 | FFA 18:3 (a-Linolenic acid, n–3 or g-Linolenic acid, n–6) | C18H30O2 | 8.31 | 5 | 18 | 10 | 15 |
| 275.2 | 275.2 | FFA 18:4 (Stearidonic acid, n–3) | C18H28O2 | 6.71 | 5 | 18 | 10 | 15 |
| 296.9 | 296.9 | FFA 19:0 (Tuberculostearic acid) | C19H38O2 | 16.20 | 5 | 14 | 10 | 14 |
| 311 | 311 | FFA 20:0 (Arachidic acid) | C20H40O2 | 17.52 | 5 | 14 | 10 | 14 |
| 309.3 | 309.3 | FFA 20:1 (cis-11-Eicosenoic acid, n–9) | C20H38O2 | 15.14 | 5 | 14 | 10 | 14 |
| 307.3 | 307.3 | FFA 20:2 (cis-11-14-Eicosadienoic acid, n–6) | C20H36O2 | 13.01 | 5 | 10 | 10 | 10 |
| 305.05 | 305.05 | FFA 20:3 (Dihomo-g-linolenic acid, n–6 or Mead acid, n–9) | C20H34O2 | 11.20 | 5 | 18 | 10 | 15 |
| 303.1 | 303.1 | FFA 20:4 (Arachidonic acid, n–6) | C20H32O2 | 9.96 | 5 | 18 | 10 | 15 |
| 300.9 | 300.9 | FFA 20:5 (Eicosapentaenoic acid, n–3) | C20H30O2 | 8.27 | 5 | 22 | 10 | 22 |
| 325.3 | 325.3 | FFA 21:0 (Heneicosanoic acid) | C21H42O2 | 18.85 | 5 | 18 | 10 | 15 |
| 339.3 | 339.3 | FFA 22:0 (Behenic acid) | C22H44O2 | 20.40 | 5 | 18 | 10 | 15 |
| 337 | 337 | FFA 22:1 (Erucic acid, n–9) | C22H42O2 | 17.94 | 5 | 18 | 10 | 15 |
| 331.3 | 331.3 | FFA 22:4 (Docosatetraenoic acid, n–6) | C22H36O2 | 12.75 | 5 | 18 | 10 | 15 |
| 329.2 | 329.2 | FFA 22:5 (Docosapentaenoic acid, n–6) | C22H34O2 | 11.52 | 5 | 16 | 10 | 16 |
| 326.95 | 326.95 | FFA 22:6 (Docosahexaenoic acid, n–3) | C22H32O2 | 9.84 | 5 | 18 | 10 | 15 |
| 353.3 | 353.3 | FFA 23:0 (Tricosanoic acid) | C23H46O2 | 21.20 | 5 | 18 | 10 | 15 |
| 367.4 | 367.4 | FFA 24:0 (Lignoceric acid) | C24H48O2 | 22.31 | 5 | 18 | 10 | 15 |
| 365.3 | 365.3 | FFA 24:1 (Nervonic acid, n–9) | C24H46O2 | 20.35 | 5 | 18 | 10 | 15 |
| 381.4 | 381.4 | FFA 25:0 (Pentacosanoic acid) | C25H50O2 | 22.97 | 5 | 18 | 10 | 15 |
| 395.4 | 395.4 | FFA 26:0 (Cerotic acid) | C26H52O2 | 24.27 | 5 | 18 | 10 | 15 |
| 409.4 | 409.4 | FFA 27:0 (Heptacosanoic acid) | C27H54O2 | 25.18 | 5 | 18 | 10 | 15 |
| 423.3 | 423.3 | FFA 28:0 (Montanic acid) | C28H56O2 | 26.19 | 5 | 19 | 10 | 19 |

# Materials and Methods

**LPC18:0 supplementation and ferrostatin-1 treatment**

Mice received LPC18:0 (20 mg/kg; Sigma-Aldrich, MO, USA, 855775P) dissolved in physiological saline by intraperitoneal injection for 21 days. Fer-1 was administered at 1 mg/kg in 0.9% saline containing 10% DMSO, 40% PEG300, and 5% Tween-80.

**Pharmacokinetic analysis of LPC18:0 after intraperitoneal administration**

To evaluate systemic LPC18:0 exposure, mice received a single intraperitoneal injection of LPC18:0 at 20 mg/kg. Blood was collected at 1, 2, 4, 6, 12, and 24 h after injection. Serum was isolated by centrifugation, stored at −80 °C, and analyzed by LC–MS/MS to determine the preliminary serum concentration–time profile of LPC18:0.

**Histological and immunofluorescence (IF) assessment of hippocampal injury**

Brain sections were stained with Nissl staining solution (Beyotime Biotechnology, Nanjing, China), differentiated in 95% ethanol for 5 min, dehydrated, and cleared in xylene. For IF staining, sections were incubated overnight at 4 °C with primary antibodies against NeuN (Proteintech, 66836-1-Ig, 1:200) or Iba-1 (Proteintech, 66827-1-Ig, 1:200), followed by goat anti-IgG secondary antibody (Bioworld, CI36131, 1:500) at 37 °C for 1 h. Sections were then washed with PBS, counterstained with DAPI (Southern Biotech, 0100-20), and imaged using an Olympus BX53 microscope.

**Detection of glutathione (GSH) and malondialdehyde (MDA) levels**

MDA and GSH levels in hippocampal tissues and cells were measured using commercial kits according to the manufacturers’ instructions (Solarbio, China; GSH, Nanjing Jiancheng Bioengineering Institute, China). Absorbance was measured using a Labserv K3 microplate reader.

**Western blot analysis**

Proteins from hippocampal tissues and cells were extracted using RIPA buffer (Beyotime, China), and concentrations were determined with a BCA assay kit. Equal amounts of protein (50 μg/sample) were separated by 10% SDS–PAGE and transferred onto PVDF membranes. After blocking, membranes were incubated overnight at 4 °C with primary antibodies against GPX4 (Proteintech, 67763-1-Ig), ALOX15 (Abclonal, A6864), and PLA2G4C (Affinity, #DF3982), followed by secondary antibodies (Proteintech, China) for 1 h at room temperature. Bands were detected using a chemiluminescent substrate kit and imaged with a ChemiDoc™ MP system.

***In vitro* blood–brain barrier (BBB) permeability assay**

An *in vitro* BBB model was established by culturing bEnd.3 cells on 0.4-μm Transwell inserts in 12-well plates until a confluent endothelial monolayer formed. Before LPC18:0 transport analysis, barrier integrity was verified using a 4-h liquid-level leakage assay, and only inserts with acceptable integrity were used. LPC18:0 was added to the upper chamber, and medium from the lower chamber was collected at the indicated time points. Transported LPC18:0 was quantified by LC–MS/MS to preliminarily assess its permeability across the in vitro BBB model.

**Transmission electron microscopy**

Fresh tissues were fixed in 2.5% glutaraldehyde for at least 4 h and post-fixed with 1% OsO₄ for more than 2 h. Samples were dehydrated through graded ethanol and acetone, embedded in Epon 816, sectioned using an RMC Boeckeler PowerTome XL ultramicrotome, and stained with uranyl acetate and lead citrate for 5 and 10 min, respectively. TEM images were acquired using a Hitachi H-7550 transmission electron microscope.

**Detection of lipid peroxidation in cells**

Cells were harvested at a density of 1.0 × 10⁶ cells/mL and stained with BODIPY™ 581/591 C11 (MCE, USA) to detect lipid peroxidation. Briefly, cells were incubated with 10 μM BODIPY dye in the dark for 30 min. The signals were measured using flow cytometry (CytoFLEX S equipped with Kaluza analysis 2.1, Beckman Colter, USA).
